# The spider mites of the genus *Eutetranychus* Banks (Acari, Trombidiformes, Tetranychidae) from Saudi Arabia: two new species, a re-description, and a key to the world species

**DOI:** 10.3897/zookeys.799.25541

**Published:** 2018-11-28

**Authors:** Muhammad Kamran, Eid Muhammad Khan, Fahad Jaber Alatawi

**Affiliations:** 1 Acarology laboratory, Department of Plant Protection, College of Food and Agriculture Sciences, King Saud University, Riyadh 11451, P.O. Box 2460, Saudi Arabia King Saud University Riyadh Saudi Arabia

**Keywords:** Key, morphological variations, new species, *
palmatus
*, phytophagous mites

## Abstract

Two new species of the genus *Eutetranychus* Banks are described and illustrated based on adult females and males, *E.spinosus***sp. n.** from *Indigoferaspinosa* Forssk (Leguminosae), *E.neotransversus***sp. n.** from *Juniperusprocera* Hochst. ex Endl. (Cupressaceae), and *E.palmatus* Attiah, 1967 is redescribed from *Washingtoniarobusta* H. Wendl. (Arecaceae). Additionally, the intraspecific morphological variations within *E.orientalis* populations, collected from 28 various host plants and 80 different localities from six regions of Saudi Arabia from 2009 to 2017, are discussed and presented. The genus *Eutetranychus* is divided into two species groups based on the presence of one seta (*orientalis* group) or two setae (*banksi* group) on coxa II. In addition, seven *Eutetranychus* species are suggested as synonyms of *E.orientalis* (Klein, 1936) and *E.papayensis* Iqbal & Ali, 2008 is considered as species inquirenda. A key to all known species of the genus *Eutetranychus* is provided.

## Introduction

The spider mites belonging to the genus *Eutetranychus* (Acari: Tetranychidae) mostly feed on shrub and tree leaves (Jeppson et al. 1975, Bolland et al. 1998) and make little webs on plant leaves (Saito 2010, Vacante 2010). Among *Eutetranychus* species, the Oriental red spider mite, *E.orientalis* (Klein) and African red spider mite, *E.banksi* (McGregor) have been recorded as major pests of citrus in many tropical and subtropical countries (Vacante 2010). Recently, *E.palmatus* Attiah was considered as a pest of date palms in Israel (Palevsky et al. 2010). Previously it has been reported from different palms (Arecaceae) from Egypt, Israel, Jordan, and Iran (Attiah 1967, Gerson et al. 1983, Kamali 1990, Ben-David et al. 2013).

The genus *Eutetryanchus* belongs to the tribe of Eurytetranychini Reck of the subfamily Tetranychinae. Banks (1917) considered *Eutetranychus* as subgenus of the *Neotetranychus* Trägårdh. Later, McGregor (1950) proposed *Eutetranychus* as valid and separated genus with type species *Tetranychusbanksi*. Baker and Pritchard (1960) provided a key to the world with eight species of *Eutetranychus*. Later, only two regional keys of *Eutetranychus* species have been constructed from India and Africa including nine and 16 species, respectively (Nassar and Ghai 1981, Meyer 1987). To date, *Eutetranychus* includes 34 nominal species, mostly reported from Africa and Asia (Migeon and Dorkeld 2006–2017). Prior to this study, no diagnostic key to those world *Eutetranychus* species is available. Only four *Eutetranychus* species viz. *E.africanus* (Tucker), *E.banksi*, *E.orientalis* and *E.palmatus* have been reported from Saudi Arabia (SA) so far (Martin 1972, Alatawi 2011).

The two species *E.orientalis* and *E.banksi* are widely distributed over the world and have been reported from approximately 223 and 84 various host plants, respectively (Bolland et al. 1998, Migeon and Dorkeld 2006–2017, Mattos and Feres 2009, Vacante 2010). Morphological variations in shape and length of dorsal setae, striation pattern between setae *d1* and *e1* and legs chaetotaxy have been reported in these two species (Baker and Pritchard 1960, Chaudhri et al. 1974, Meyer 1974, Meyer 1987, Khanjani et al. 2017). Because of such variations, some *Eutetryanchus* species have been synonymized with *E.orientalis* (Baker and Pritchard 1960, Meyer 1987) and others with *E.banksi* (Pritchard and Baker 1955, Bolland et al. 1998).

The aims of the present study were to explore *Eutetranychus* species from Saudi Arabia, to develop a key to the world species of this genus and to discuss the morphological intraspecific variations in *E.orientalis* populations collected from different hosts and localities from Saudi Arabia. In this study, two new species of *Eutetranychus*; *E.spinosus* sp. n. and *E.neotransversus* sp. n. are described and illustrated based on adult females and males (Figs 1–30). Also, *E.palmatus* is redescribed and illustrated based on adult female and male (Figs 31–46) because its original description was brief and incomplete from date palm trees in Egypt (Attiah 1967). Two previous recorded species, *E.africanus* and *E.banksi*, from SA were not found in this comprehensive collection. The intraspecific morphological variations within *E.orientalis* populations collected from 28 various host plants and 80 different localities in six regions of SA during 2009 to 2017, are discussed and presented (Figs 47A–H, 48, 49).

## Materials and methods

*Eutetranychus* spider mites were collected from diverse host plants from different localities in six regions (Al–Ula, Madina, Nijran, Riyadh, Tabuk, and Taif) of SA during 2009–2017. The mite specimens were collected by shaking the aerial parts of plants over a white piece of paper. The mites moving on paper were picked with camel hair brush and preserved in small vials containing 70% alcohol, then mounted in Hoyer’s medium under a stereomicroscope (SZX10, Olympus, Tokyo, Japan). The specimens were examined and identified under a phase contrast microscope (BX51, Olympus®, Japan) using keys and available literature. Different mite body parts were pictured by using an auto-montage software system (Syncroscopy, Cambridge, UK) and then drawn with Adobe Illustrator (Adobe SystemInc., San Jose, CA, USA). All measurements are given in micrometers. The lengths of the legs were measured from the base of the trochanter to the tip of tarsus. The measurements are presented for the holotype followed by the range of paratypes in parenthesis. The morphological terminology used in this study follows that of Lindquist (1985). All collected specimens including type specimens of the new species have been deposited at King Saud University Museum of Arthropods (**KSMA**, Acarology section), Department of Plant Protection, College of Food and Agriculture Sciences, King Saud University, Riyadh, SA.

### Family TETYRANYCHIDAE Donnadieu

#### Subfamily Tetranychinae Berlese

##### Tribe Eurytetranychini Reck

###### Genus Eutetranychus

Taxon classificationAnimaliaProstigmataTetranychidae

Banks

Neotetranychus (Eutetranychus) Banks, 1917: 197.
Anychus McGregor, 1919: 644.
Eutetranychus Banks, McGregor 1950: 267.

####### Type species.

*Tetranychusbanksi* McGregor, 1914.

####### Diagnosis.

Based on Meyer 1987. The genus *Eutetranychus* can be recognized by the combination of following characters: Propodosomal setae three pairs (*v2*, s*c1* and *sc2*); opisthosomal setae 10 pairs (*c1−3, d1−2, e1−2, f1−2, h1*); setae *f1* either normally or widely spaced; anal setae (*ps_1-2_*) and para anal setae (*h_2-3_*) each two pairs; empodium absent or reduced to small rounded tiny knob; true claws pad-like; tarsi I with two or three solenidia (two solenidia closely associated with fastigial setae *ft*), tarsi II with one or two solenidia; coxa II with either one or two setae.

###### Species group *banksi*

**Diagnosis.** Coxa II with two setae.

####### Eutetranychus spinosus sp. n.

Taxon classificationAnimaliaProstigmataTetranychidae

http://zoobank.org/DA81A602-25EC-458E-86B9-C029405EDEC4

Figures 1 , 2 , 3 , 4−7 , 8 , 9 , 10 , 11 , 12–15

######## Diagnosis.

(Based on female). Dorsal body setae long, slender, serrate, all set on small tubercles except *v2* and *sc1*, dorsocentral setae *c1*, *e1* and *f1* longer than the distance between their base and the bases of next consecutive setae; setae *c1* and *f1* shorter than distances between *c1*−*c1* and *f1*−*f1* respectively, setae *e1* almost as long as distance *e1*−*e1*; dorsum with simple striae except area anterior to setae *sc1* with lobed striae, striae between setae *d1* “V” shaped, genua and tibiae I−IV 5−5−3−3; 9(1)−7−8−8, respectively.

######## Description.

**Figure 1.**
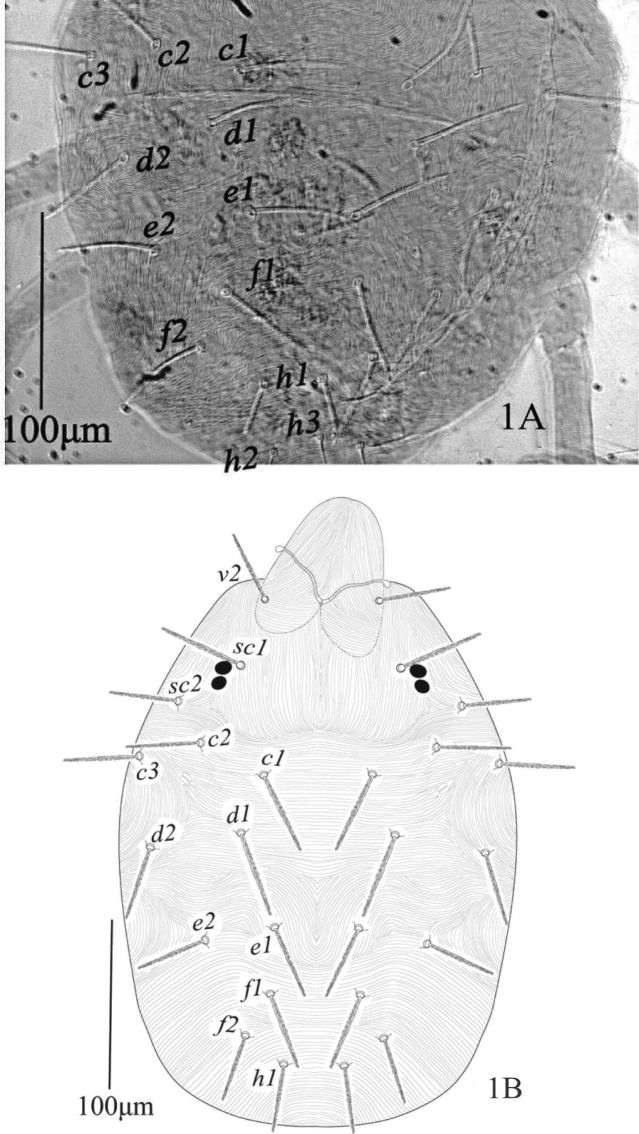
*Eutetranychusspinosus* sp. n. Female, Dorsum (**A, B**).

**Figure 2.**
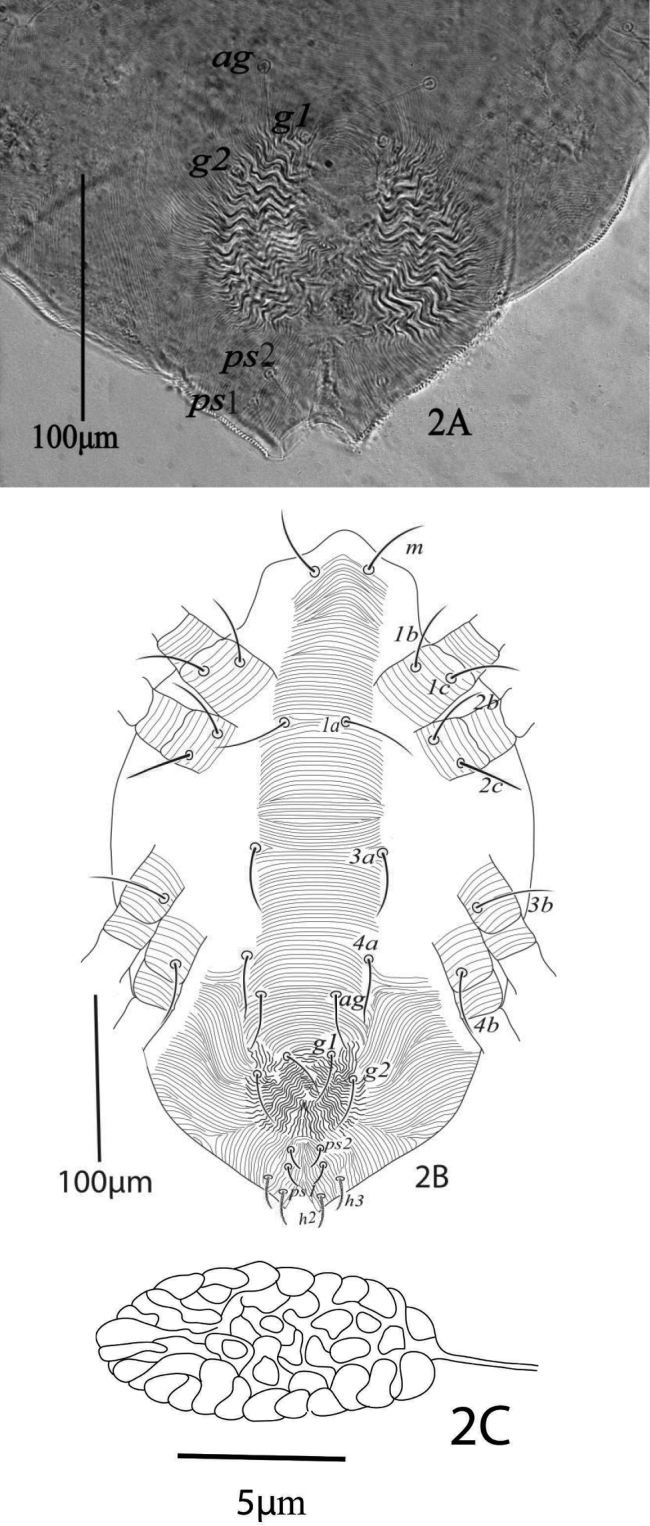
*Eutetranychusspinosus* sp. n. Female **A** genito-anal region **B** Venter **C** Spermatheca.

**Figure 3.**
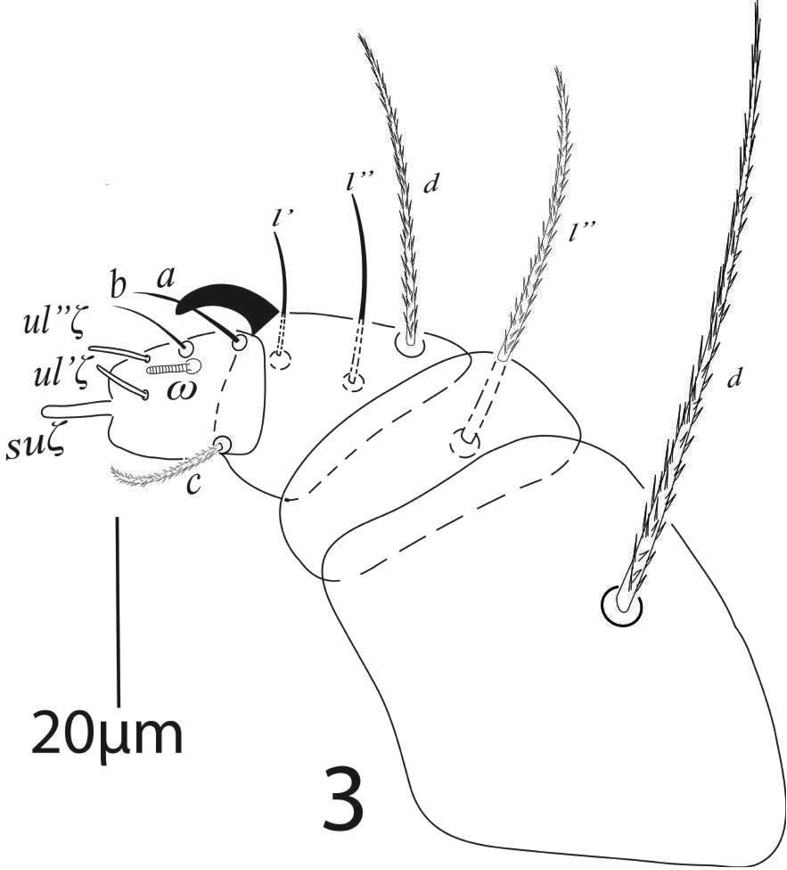
*Eutetranychusspinosus* sp. n. Female, Palp.

**Figures 4–7.**
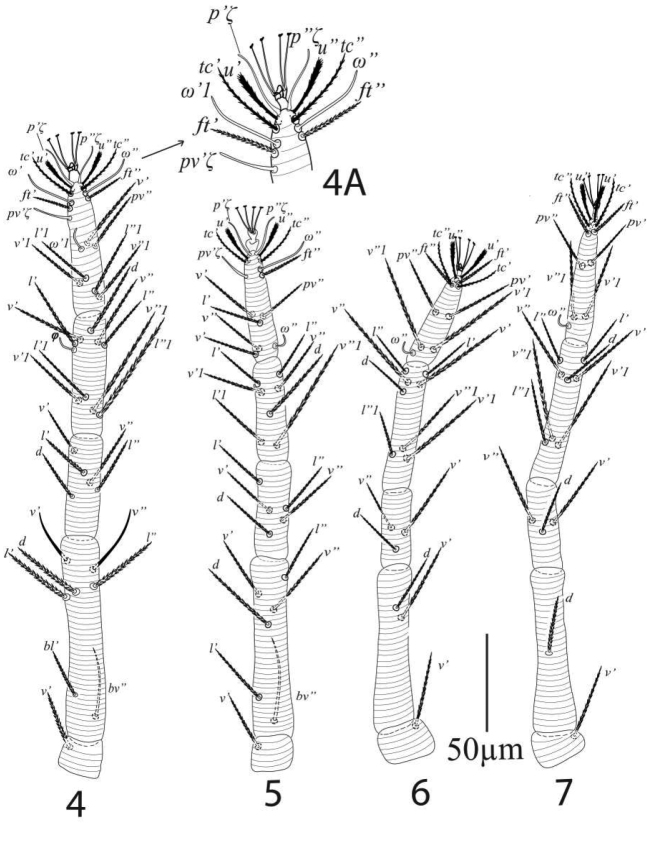
*Eutetranychusspinosus* sp. n. Female, **4** Leg 1 **4A** Leg 1 tarsus **5** Leg 2 **6** Legs 3 **7** Leg 4.

**Female** (n = 12) (Figures 1–7).

*Body* oval, color in life greenish yellow. Length of body (excluding gnathosoma) 315 (312−325), (including gnathosoma) 396 (390−405), maximum width 221 (218−231).

*Dorsum* (Figure 1). Propodosoma medially with longitudinal striae; hysterosoma medially with transverse striae except area between setae *d1* and *e1* forming a V-shaped pattern; dorsal striae simple except anterior of setae *sc1* with small lobes; all dorsal setae slender, serrated and sub-equal in length, setae *sc2* and hysterosomal setae set on small tubercles; setae *v2* almost reaching 2/3 to the distance *v2−v2*; dorsocentral setae *c1*, and *f1* reaching to past bases of next consecutive setae; setae *e1* almost as long as distance *e1*−*f1*; setae *f1* slightly more widely spaced than *e1*. Length of dorsal setae: *v2* 44 (41−45), *sc1* 56 (53−58), *sc2* 44 (42−46), *c1* 50 (47−52), *c2* 44 (42−45), *c3* 46 (45−48), *d1* 53 (51−55), *d2* 47 (45−49), *e1* 47 (46−48), *e2* 44 (42−45), *f1* 48 (47−50), *f2* 42 (40−44), *h1* 42 (40−44); distance between dorsal setae: *v2−v2* 66 (63−68), *sc1−sc1* 95 (91−97), *sc2−sc2* 165 (162−170), *c1−c1* 63 (60−67), *c2−c2* 147 (142−150), *c3−c3* 200 (195−210), *d1−d1* 95 (92−97), *d2−d2* 189 (186−191), *e1−e1* 53 (50−55), *e2−e2* 158 (155−160), *f1−f1* 58 (55−64), *f2−f2* 79 (77−82), *h1−h1* 34 (33−36), *v2−sc1* 42 (40−44), *sc1−sc2* 50 (48−53), *sc2−c3* 45 (44−47), *sc2−c2* 53 (52−55), *sc2−c1* 95 (93−98), *c1−c2* 44 (42−46), *c2−c3* 33 (32−36), *c1−d1* 37 (36−39), *c2−d2* 68 (66−70), *d1−e1* 51 (49−52), *d2−e2* 59 (57−60), *e1−f1* 42 (40−44), *e2−f2* 48 (46−50), *f1−h1* 39 (36−41), *f2−h1* 25 (22−27).

*Venter* (Figure 2). Ventral cuticle medially with transverse striae from setae *1a* to setae *g1*, length of ventral setae: *1a* 50 (48−53), *3a* 42 (41−44), *4a* 44 (43−46), *1b* 42 (40−45), *1c* 45 (41−46), *2b* 42 (40−43), *2c* 39 (37−41), *3b* 47 (45−49), *4b* 42 (41−43); distance between intercoxal and coxae setae: *1a−1a* 37 (35−38), *1b−1c* 17 (17−18), *3a−3a* 79 (77−80), *4a−4a* 75 (73−78); aggenital setae: *ag* 37 (37−38), *ag−ag* 54 (49−57); genital setae: *g1* 31 (30−33), *g2* 32 (31−34), *g1−g1* 26 (23−28), *g2*−*g2* 60 (57−61); anal setae two pairs: *ps1* 12 (12−13), *ps2* 12 (11−13), *ps1−ps2* 8 (8−9), *ps1−ps1* 21 (20−23), *ps2−ps2* 17 (17−18); para-anal setae two pairs *h2* 21 (19−22), *h3* 19 (18−19), *h2−h2* 25 (23−26), *h3−h3* 45 (40−45). All ventral setae simple except *h2* and *h3* barbed. Spermatheca elongated and sacculus terminally rounded (Figure 2C).

*Gnathosoma* (Figure 3). Subcapitular setae *m* 41 (39−43), *m*−*m* 31 (29−32) (Figure 2). Palp femur and genu each with one setae *d* 51 (49−55), *l*” 43 (40−45); palp tibia with three setae *d* 34 (31−34), *l*” 21 (20−22), *l*’ 13 (13−14) and a palp tibial claw; palp tarsus 17(17−18) long, 13 wide, with 3 simple setae *a* 13 (12−13), *b* 9 (9−10), *c* 13 (13−14), 3 eupathidia *su*ζ 7.5 (7.5−8), width 1.3 (1−1.5), *ul*’’ζ = *ul*’ζ 6.5 (6.5−7) width 1.2 (1−1.3), a solenidion ω 5 long width 2 (1.8−2.3) (Figure 3). Stylophore anteriorly rounded; peritremes ending with simple bulb (Figure 1).

*Legs* (Figures 4–7). Length of legs I−IV (trochanter to pretarsus): 320 (313−323), 263 (255−270), 294 (288−300), 336 (325−340) respectively; leg I: trochanter 21 (19−21), femur 105 (100−109), genu 53 (51−55), tibia 63 (60−68), tarsus 79 (74−82); leg II: trochanter 16 (15−17), femur 95 (91−99), genu 42 (41−44), tibia 53 (51−55), tarsus 58 (54−60); leg III: trochanter 17 (17−18), femur 86 (84−90), genu 44 (42−47), tibia 71 (68−75), tarsus 76 (74−79); leg IV: trochanter 19 (18−20), femur 105 (102−108), genu 47 (45−50), tibia 79 (76−83), tarsus 86 (83−90); legs chaetotaxy I−IV (eupathidia and solenidia in parenthesis): coxae 2−2−1−1; trochanters 1−1−1−1; femora 7−6−2−1; genua 5−5−3−3; tibiae 9(1)−7−8−8; tarsi 12(3ζ, 3ω)−10(3ζ, 2ω)−10(1ω)−10(1ω).

**Male** (n = 3) (Figures 8–15).

Length of *body* (excluding gnathosoma) 300−310, (including gnathosoma) 350−361, maximum width 237−246.

**Figure 8.**
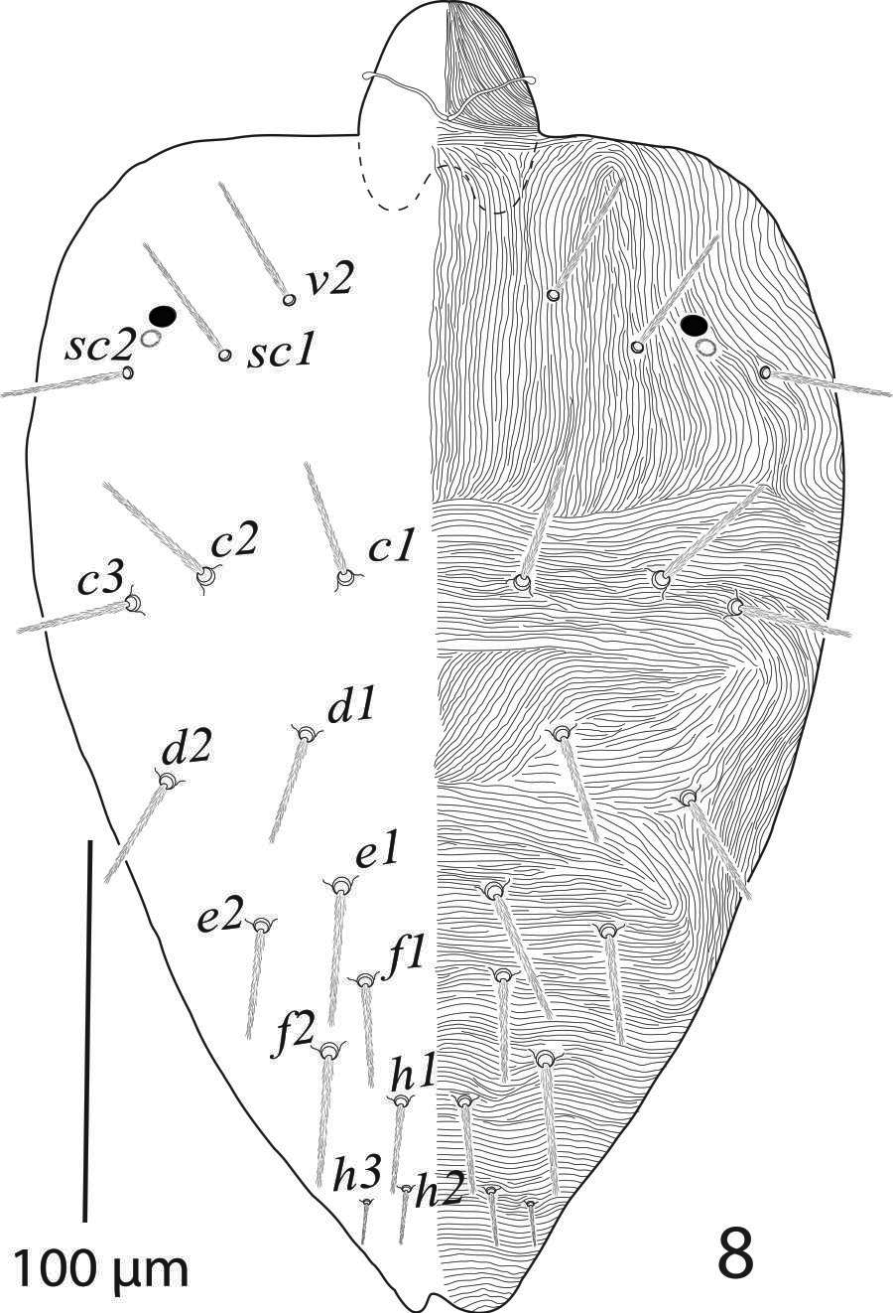
*Eutetranychusspinosus* sp. n. Male, Dorsum.

*Dorsum* (Figure 8). Propodosoma medially with longitudinal striae; hysterosoma medially with transverse to irregular striae and forming a V-shaped pattern in between setae *d1* and *e1*; all dorsal body setae slender, serrated and sub-equal in length, hysterosomal setae set on small tubercles. Length of dorsal setae: *v2* 34−38, *sc1* 35−40, *sc2* 32−37, *c1* 31−35, *c2* 36−39, *c3* 32−35, *d1* 29−32, *d2* 33−35, *e1* 33−38, *e2* 32−35, *f1* 28−33, *f2* 33−37, *h1* 23−26, *h2* 15−19, *h3* 13−16; distance between dorsal setae: *v2−v2* 45−50, *sc1−sc1* 73−80, *sc2−sc2* 162−173, *c1−c1* 44−47, *c2−c2* 112−120, *c3−c3* 150−162, *d1−d1* 65−70, *d2−d2* 136−151, *e1−e1* 37−41, *e2−e2* 90−100, *f1−f1* 35−40, *f2−f2* 55−60, *h1−h1* 14−17, *h2−h2* 10−13, *h3−h3* 32−36, *v2−sc1* 28−32, *sc1−sc2* 32−33, *sc2−c3* 55−60, *sc2−c2* 45−50, *sc2−c1* 55−60, *c1−c2* 30−34, *c2−c3* 22−26, *c1−d1* 39−43, *c2−d2* 50−56, *d1−e1* 42−46, *d2−e2* 35−40, *e1−f1* 20−24, *e2−f2* 35−40, *f1−h1* 36−41, *f2−h1* 25−30.

**Figure 9.**
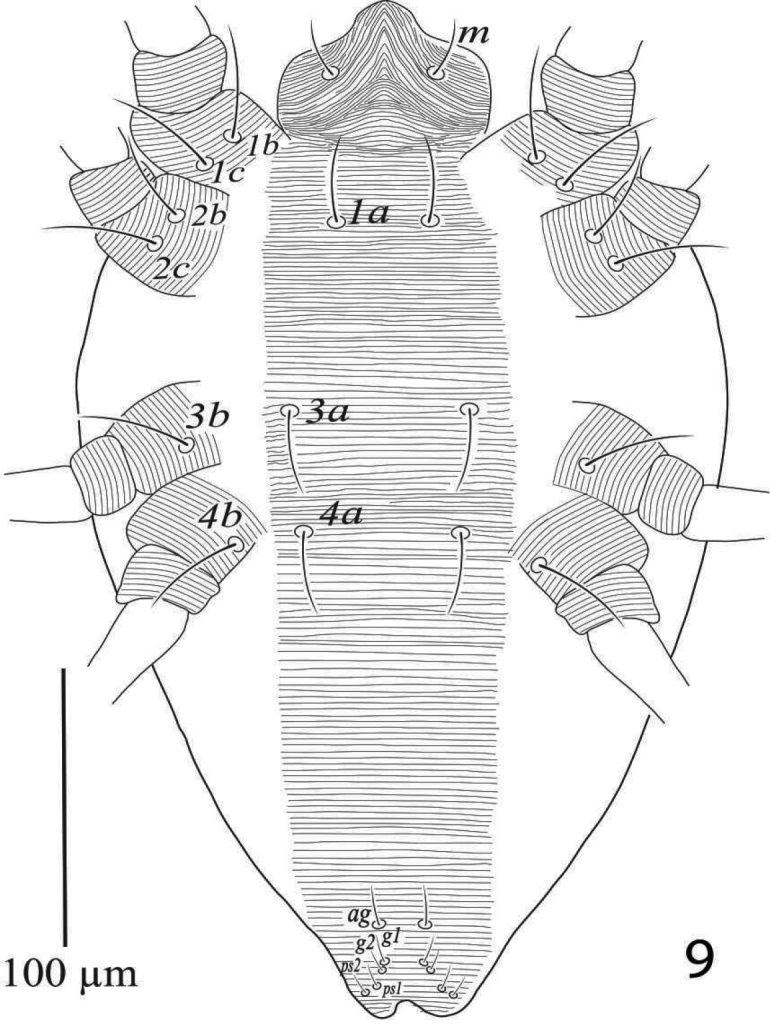
*Eutetranychusspinosus* sp. n. Male, Venter.

Venter (Figure 9). Area between setae *1a* to *ag* with transverse striae; length of ventral setae: *1a* 30−33, *3a* 31−35, *4a* 32−37, *1b* 40−45, *1c* 40−45, *2b* 30−33, *2c* 37−41, *3b* 36−39, *4b* 36−41; distance between setae: *1a−1a* 30−36, *1b−1c* 17−18, *3a−3a* 62−68, *4a−4a* 51−58; aggenital setae: *ag* 15−18, *ag−ag* 49−54; genital setae: *g1* 9−13, *g2* 8−10, *g1−g1* 14−17, *g2*−*g2* 15−18; anal setae two pairs: *ps1* 10−12, *ps2* 11−13, *ps1−ps2* 3−4, *ps1−ps1* 24−28, *ps2−ps2* 22−26.

**Figure 10.**
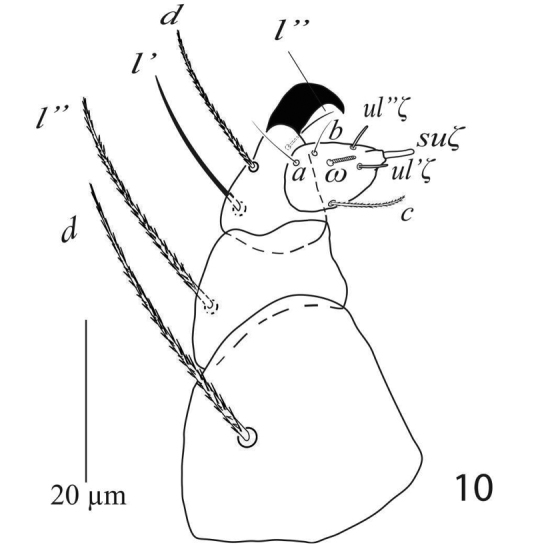
*Eutetranychusspinosus* sp. n. Male, Palp.

*Gnathosoma* (Figure 10). Subcapitular setae *m* 30−34, *m*−*m* 27−31 (Figure 9); palp femur and genu each with one setae *d* 35−41, *l*” 31−35; palp tibia with three setae *d* 18−22, *l*” 21−25, *l*’ 13−14 and a palp tibial claw; palp tarsus 11−14 long, 10 wide, with 3 simple setae *a* 9−11, *b* 7−10, *c* 8−10, 3 eupathidia *su*ζ 6.5−7, width 0.9 (0.8−1), *ul*ζ = *ul*’ζ 6−7, width 0.8 (0.7−1) a solenidion ω 4 long width 1 (0.9−1.2) (Figure 10). Stylophore anteriorly rounded; peritremes ending with simple bulb (Figure 8).

**Figure 11.**
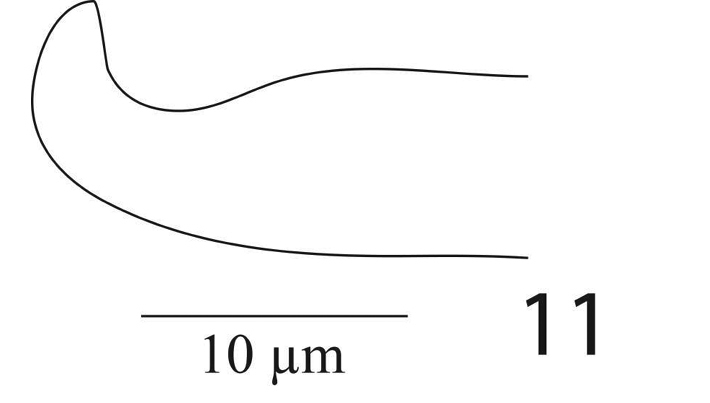
*Eutetranychusspinosus* sp. n. Male, Aedeagus.

*Aedeagus* (Figure 11) Aedeagus bends dorsad at an angle of 90°; the bent portion narrowly rounded toward tip and blunt distally, shaft 18 long, 7 wide, bent portion 3 long.

**Figures 12–15.**
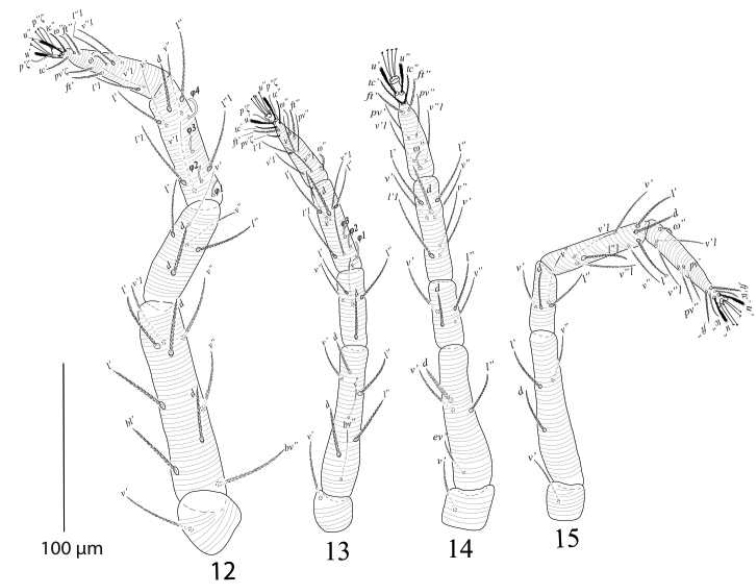
*Eutetranychusspinosus* sp. n. Male, **12** Leg 1 **13** Leg 2 **14** Leg 3 **15** Leg 4.

*Legs* (Figures 12–15). Length of legs I−IV (trochanter to pretarsus): 313−328, 235−250, 263−280, 278−295 respectively; legs I−IV chaetotaxy (eupathidia and solenidia in parenthesis): coxae 2−2−1−1; trochanters 1−1−1−1; femora 8−7−4−3; genua 5−5−4−4; tibiae 8(4)−7(3)−8−8; tarsi 11(3ζ, 2ω)−11(3ζ, 2ω)−10(1ω)−10(1ω).

######## Immature stages.

unknown.

######## Etymology.

The species name is derived from name of the host plant species, *Indigoferaspinosa*, of which type specimens were collected.

######## Type material.

Holotype female and four paratype females, *Indigoferaspinosa* (Leguminosae), Al- Shifa road, Taif, 21°05.824'N, 040°19.111'E, elevation 2102 m, 11 Oct 2016, leg. M Kamran and M Rehman; five paratype females, *Indigoferaspinosa* (Leguminosae), As Sayl Saghir, Taif, 21°30.521'N, 040°28.202'E, elevation 1516 m, 10 Sept 2017, leg. Eid M Khan and M Rehman; two paratype females, *Indigoferaspinose* (Leguminosae), Al Sayl Kabeer, Taif, 21°37.371'N, 040°24.212'E, elevation 1240 m, 15 Sept 2017, leg. Eid M Khan and M Rehman.

######## Remarks.

*Eutetranychusspinosus* sp. n. belongs to the *banksi* species group. It closely resembles *E.namibianus*Meyer 1987 because both have same legs chaetotaxy (Table 1) and dorsal striae pattern. However, the new species differs from *E.namibianus* by all dorsal setae slender, much longer, mostly longer than the distance between their base and the bases of next consecutive setae vs. all dorsal setae sub-spatulate, small, far behind the bases of next consecutive setae, setae *c1* and *e1* crossing the bases of next consecutive setae vs. reaching less than half distance to the bases of setae next in line and all hysterosomal setae set on strong tubercles vs. only some setae on opisthosoma set on tubercles in *E.namibianus*. The new species also resembles *E.acaciae*Miller 1966 because both have all dorsal setae slender, much longer, and mostly longer than the distance between their base and the bases of next consecutive setae. The new species can be separated from *E.acaciae* by setae *f1* slightly more widely spaced as setae *e1* vs. *f1* two time more widely spaced as compare to *e1*, differences in legs chaetotaxy, genua I−IV with 5−5−4/3−3 vs. 3−3−1−1 and femora II & III with 6 & 2 vs. 4 & 3, respectively in *E.acaciae*.

**Table 1.** Legs chaetotaxy of world species of the genus *Eutetranychus* (including new species).

Species	Femora I–IV	Genua I–IV	Tibiae I–IV	Tarsi I–IV	Reference
**Species group *orientalis***					
*neotransversus* sp. n.	5-4-2-1	4-4-1-2	6(1)-5-4-4	15(2)-14(1)-10(1)-10(1)	Present study
* bilobatus *	8-5-4-1	5-5-2-2	9(1)-6-6-7	15(2)-13(2)-10-10	Nassar and Ghai 1981
* caricae *	7-6-2-1	5-4-1-1	8-5-5-5	15(2)-12 (1)-10(2)-10(1)	Nassar and Ghai 1981
* citri *	–	–	9(1)-5	–	Attiah 1967
* maximae *	8-6-3-1	5-5-2-2	9(1)-6-5-7	15(2)-13(2)-10(1)-9(1)	Nassar and Ghai 1981
* mirpuriensis *	8-6-3-2	5-5-2-2	10-6-6-7	14-12-11-11	Chaudhri et al. 1974
* nagai *	8-5-3-1	5-5-2-2	9(1)-6-6-7	15(2)-13(1)- 9(2)-10 (1)	Nassar and Ghai 1981
* orientalis *	8-6-3/4 -1/2	5-5-2-2	9(1-4)-6(0-2)-6(0-1)-7	15(3)-13(1-2)-10(1)-10(1)	Meyer 1974
	8-6-4-2	5-5-2-2	9-7-6-7	15-13-11-11	Chaudhri et al. 1974
	8-7/6-3/4-1/2	5-5-2-2	9(1)-6-6-7	15(3)-13(1)-10(1)-10(1)	Khanjani et al. 2017
	8/7-7/6/5-4/3-1/2	5-5-2-2	9/8(1)-7/6-6/5-7/6	15(3)-13(1)-10(1)-10(1)	Present study
* palmatus *	–	–	9(1)	–	Attiah 1967
	8-6-2-1	5-5-2-2	9(2)-6(2)-6-7	15(3)-13(2)-10(1)-10(1)	Meyer 1987
	8-7-4-1	5-5-2-2	9(1)-6-6-7	15(3)-13(1)-10(1)-10(1)	Present study
* pantopus *	–	–	9(1)-5	–	Meyer 1974
* pyri *	–	–	9(1)-5	–	Attiah 1967
* transverstriatus *	7-7-4-3	4-4-2-2	10-6-6-6	10-10/9-10(1)-10(1)	Smiley and Baker 1995
*fici	8-6-3-2	5-5/6-2-2	9(1)-6-6-7	15(1-3)-13(1)-10(1)-10(1)	Meyer 1987
*phaseoli	8-7-3-1	5-5-2-2	9(1)-6-6-7	15(3)-13(2)-10(1)-10	Nassar and Ghai 1981
* *pruni*	8-7-3-1	5-5-2-2	9(1)-6-6-7	12(3)-11(1)-8(1)-8(1)	Smiley and Baker 1995
*ricinus	8-7-4/3-2	5-5-2-2	9/8(1)-6-6-7	15(1)-10(1)-10(1)-10(1)	Smiley and Baker 1995
*sanaae	8-7-4/3-1	5-5-2-2	9(1)-7/6-6-7	11(1)-11(2)-10(1)-10(1)	Smiley and Baker 1995
*guangdongensis	Mentioned in original description same as *E.orientalis*				Ma and Yaun 1982
*xianensis					Ma and Yaun 1982
**Species group *banksi***					
*spinosus* sp. n.	8/7-6-2-1	5-5-4/3-3	9(1)-7-8-8	15(2)-13(2)-10(1)-10(1)	Present study
* acaciae *	6/7-4-3-1	3-3-1-1	8(1)-4/5-3-5	13(4)- 12(3)-10(1)-10(1)	Based on pictures send by Dr. Owen D. Seeman
* africanus *	8-6-3-1	5-5-2-2	9(1)-6-6-7	15(2/3)-13(1)-10(1)-10(1)	Meyer 1987
*** anitae ***	9-6-4-3	5-5-3-2	9(4)-7(2)-6/7-7	13(3)-12(2)-11(1)-10(1)	By personal communication with Dr. Elizeu Castro
*** banksi ***	6/7-4/6-2-1	4-4-2-2	9(1)-6-4/5-5/6	14(2)-12(2)-10(1)-10(1)	Mattos and Feres 2009
*** bredini ***	8-7-4-1	5-5-2-2	9(1)-5-5	–	Baker and Pritchard 1960
*** carinae ***	8-6-2-1	5-5-2-2	9(1)-6-5-6	15(2)-13(1)-10(1)-10(1)	Meyer 1987
*** clastus ***	8-6-3-1	5-5-2-2	9(1)-5-5-6	15(3)-13(1)-10(1)-10(1)	Meyer 1987
*** concertativus ***	7-6-2-2	5-5-5-3	9(1)-7-8-8	15(2)-13(2)-10(1)-10(1)	Meyer 1987
*** cratis ***	8-6-3-2	3-3-2-2	6(1)-5-4-5	14(3)-13(1)-10-10(1)	Meyer 1987
*** eliei ***	8-6-2-1	5-5-2-2	9(1)-6-5-6	15(1)-13(1)-10(1)-10(1)	Meyer 1987
*** enodes ***	8-6-3-1	5-5-2-2	9(1)-6-5-7	15(2)-13(2)-10(1)-10(1)	Meyer 1987
*** namibianus ***	7-6-2-1	5-5-3-3	9(1)-7-8-8	15(2)-13(1)-10(1)-10(1)	Meyer 1987
*** nomurai ***	6-6-2-1	5-5-2-2	9(1)-8/7-8/7-8/7/9	12/13/14(3)-12/13(1/2)-12/13(1/2)-10(1)	Flechtmann 1997
*** rhusi ***	7-6-2-1	5-5-3-3	9(1)-7-7-8/7	15(2)-13(1)-10(1)-10(1)	Meyer and Ueckermann 1988
*** swazilandicus ***	8-6-2-2	5-5-3-3	9(1)-7-8-8	15(3)-13(2) - 10(1) - 10(1)	Meyer 1987

* Suggested synonyms of *E.orientalis* All missing characters/values in the table were not described or illustrated in the original descriptions/re-descriptions.

###### Species group *orientalis*

**Diagnosis.** Coxa II with one seta.

####### Eutetranychus neotranversus sp. n.

Taxon classificationAnimaliaProstigmataTetranychidae

http://zoobank.org/50D5AC16-EE8D-4508-8A29-103B8FA68D34

Figures 16 , 17 , 18 , 19−22 , 23 , 24 , 25 , 26 , 27–30

######## Diagnosis (Female).

Dorsal body setae slender and serrate, all set on small tubercles; hysterosoma medially with transverse striae; propodosoma with lobed striae, hysterosomal striae simple (without lobes); stylophore slightly notched anteriorly; leg I shorter than body length; femora, genua, tibiae and tarsi I−IV: 5−4−2−1; 4−4−1−2; 6 (1)−5−4−4; 12(3ζ, 2ω)−11(3ζ, 1ω)−10(1ω)−10(1ω), respectively.

######## Description.

**Figure 16.**
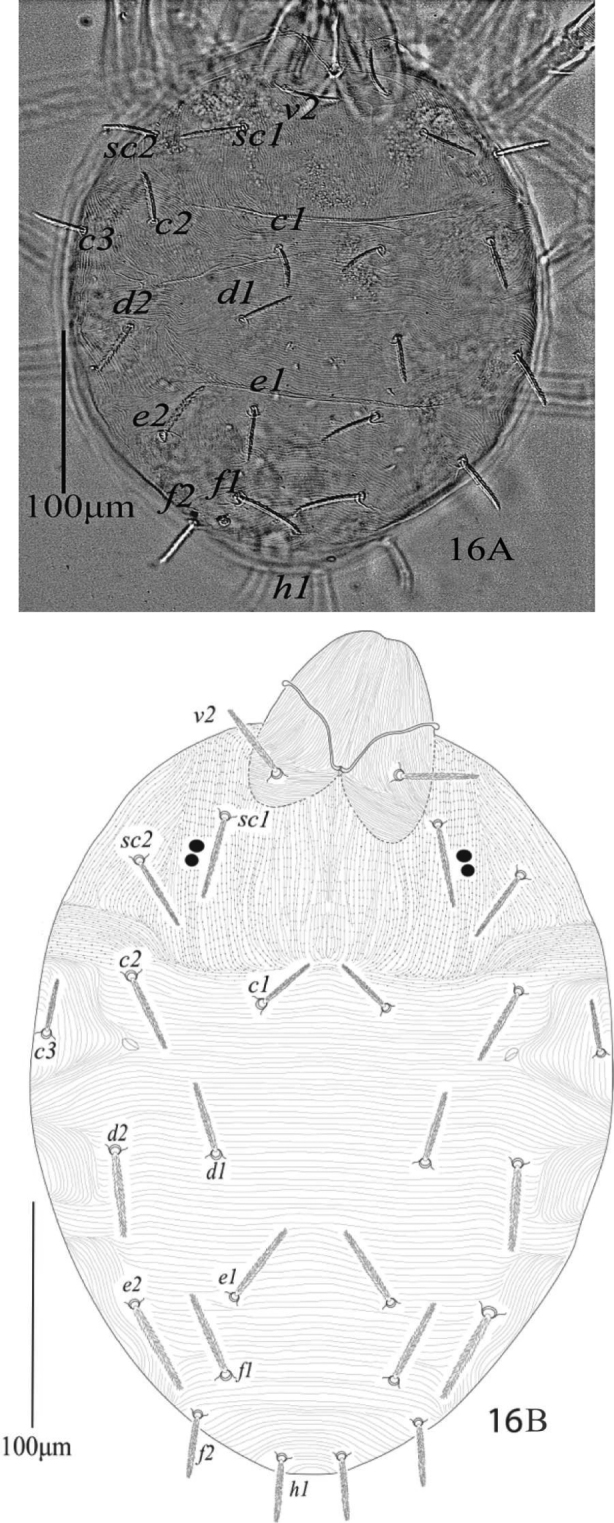
*Eutetranychusneotransversus* sp. n. Female, Dorsum (**A, B**).

**Figure 17.**
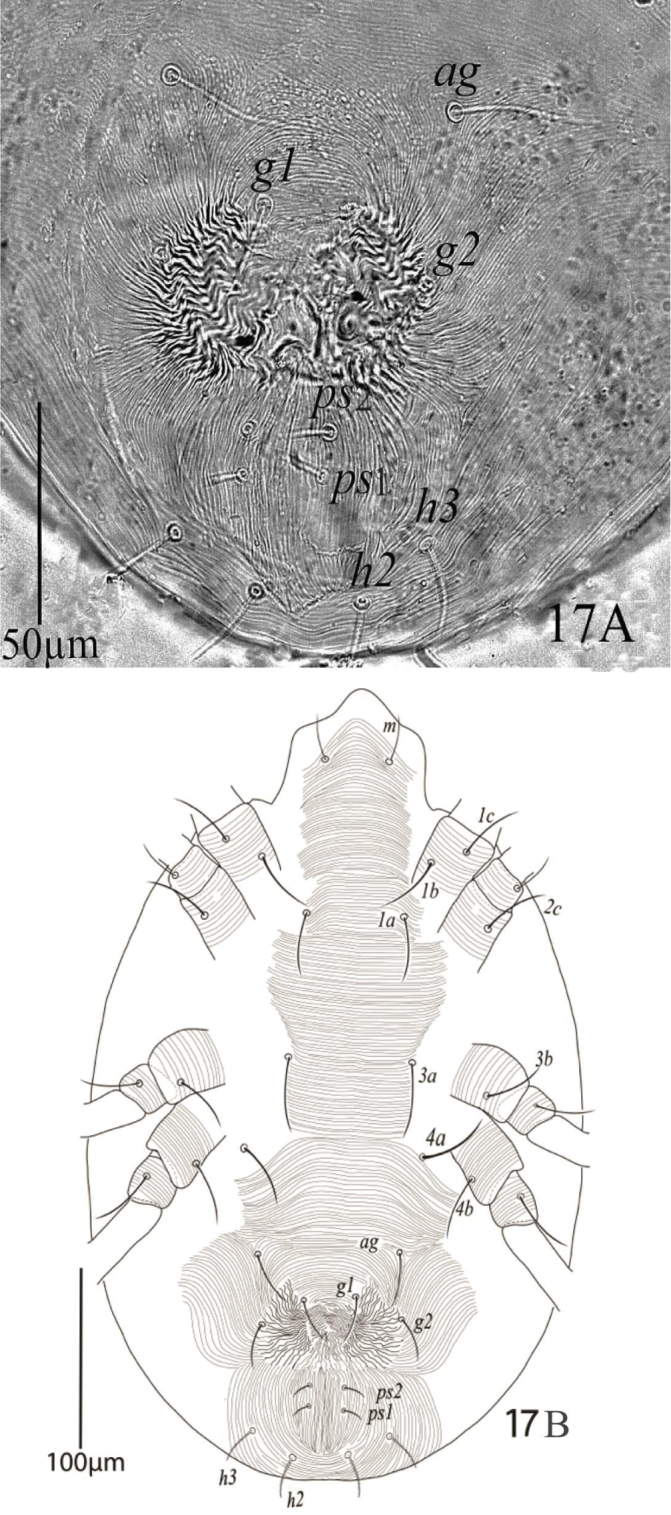
*Eutetranychusneotransversus* sp. n. Female, **A** Genito-anal region **B** Venter.

**Figure 18.**
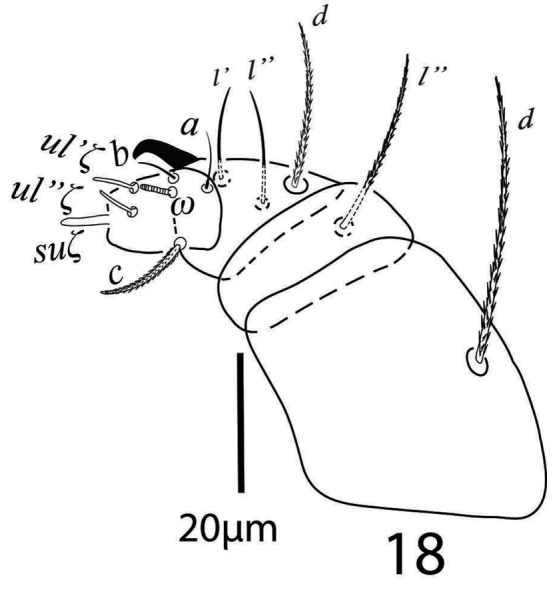
*Eutetranychusneotransversus* sp. n. Female, Palp.

**Figures 19–22.**
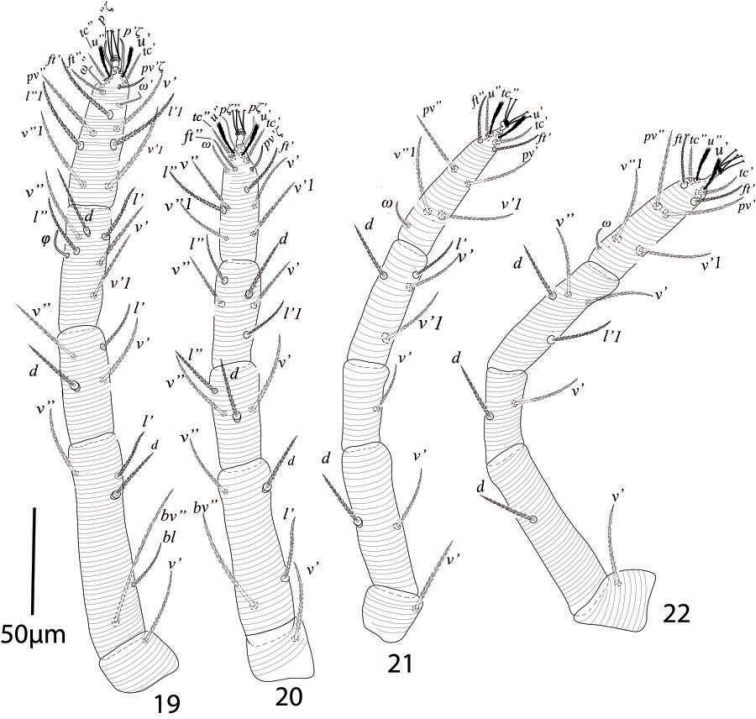
*Eutetranychusneotransversus* sp. n. Female, **19** Leg 1 **20** Leg 2 **21** Leg 3 **22** Leg 4.

**Female** (n = 8) (Figures 16–22). *Body* oval; length of body (excluding gnathosoma) 347 (340−355), (including gnathosoma) 425 (415−430) and maximum width 263 (255−270).

*Dorsum* (Figure 16). Propodosoma medially with longitudinal striae, propodosoma with lobed striae, hysterososma medially with transverse striae, hysterosoma with simple striae; dorsal body setae slender and serrate, all dorsal setae with small tubercles, setae *v2* reaching about two third to the distance *v2−v2*, reaching to the base of setae *sc1*; most hysterosomal setae distinctly shorter than distances of setae next row distance *f1−f1* almost as long as *d1*−*d1* but more widely spaced than *c1*−*c1* and *e1*−*e1*. Length of dorsal setae: *v2* 34 (32−36), *sc1* 37 (36−38), *sc2* 32 (32−33), *c1* 24 (24−25), *c2* 36 (35−36), *c3* 23 (22−23), *d1* 33 (33−35), *d2* 38 (36−38), *e1* 37 (36−38), *e2* 42 (40−44), *f1* 39 (36−41), *f2* 26 (24−27), *h1* 28 (27−29); distances between dorsal setae: *v2−v2* 53 (51−55), *sc1−sc1* 95 (93−96), *sc2−sc2* 163 (160−165), *c1−c1* 58 (55−59), *c2−c2* 168 (160−170), *c3−c3* 263 (260−268), *d1−d1* 95(93−97), *d2−d2* 179 (174−185), *e1−e1* 63 (61−66), *e2−e2* 168 (163−170), *f1−f1* 73 (70−75), *f2−f2* 100 (97−102), *h1−h1* 26 (25−28), *v2−sc1* 27 (26−29), *sc1−sc2* 43 (42−45), *sc2−c3* 89 (87−90), *sc2−c2* 58 (57−59), *sc2−c1* 86 (85−87), *c1−c2* 53 (52−55), *c2*−*c3* 50 (50−52), *d1−d2* 48 (47−49), *e1−e2* 47 (46−48), *f1−f2* 21 (20−22), *c1−d1* 63 (60−64), *c2*−*d2* 74 (73−75), *d1−e1* 68 (66−69), *d2−e2* 74 (73−75), *e1−f1* 42 (41−43), *e2−f2* 53 (52−54), *f1−h1* 40 (39−41), *f2−h1* 37 (36−38).

*Venter* (Figure 17). Area between setae *1a*−*g1* with transverse striae. Length of ventral setae: *1a* 37 (34−38), *3a* 39 (39−40), *4a* 40 (41−42), *1b* 44 (41−44), *1c* 43 (42−44), *2c* 37 (35−38), *3b* 36 (35−38), *4b* 36 (33–37); distances between intercoxal and coxae setae: *1a−1a* 53 (51−54), *3a−3a* 68 (66−70), *4a−4a* 95 (92−97); agential setae *ag* 42 (37−43), *ag−ag* 85 (83−86); genital setae: *g1* 33 (30−33), *g2* 32 (30−32), *g1−g1* 25 (24−26), *g2−g2* 81 (76−85); anal setae two pairs: *ps1*= *ps2* 14 (13−15), *ps1−ps1* 23 (21−23), *ps2−ps2* 23 (22−23); para−anal setae two pairs: *h2* 28 (27−28), *h3* 26 (26−28), *h2−h2* 31 (29−34), *h3−h3* 75 (72−77); all ventral setae simple except *h2* and *h3* slightly barbed. Spermatheca not clear.

*Gnathosoma* (Figure 18). Subcapitular setae *m* 23 (22−25), *m*−*m* 42 (39−44) (Figure 17). Palp femur and genu each with one setae, *d* 40 (39−44), *l*” 42 (40−43); palp tibia with three setae *d* 16 (14−17), *l*” 23 (21−25), *l*’ 23 (21−25) and a palp tibial claw; palp tarsus 16 (16−17) long, 11 (11−12) wide at base, with three setae *a* 7 (7−8), *b* 7 (6−7) both simple, *c* 13 (12−13) slightly barbed, three eupathidia *su*ζ’’ 7 (6.5−7) long, 1.60 wide, *ul*’’ζ 6, *ul*’ζ 6 and one solenidion ω 5 width 1.7 (1.5−2) (Figure 18). Stylophore anteriorly slightly notched; peritreme ending with a simple bulb (Figure 16).

*Legs* (Figures 19–22). Length of legs I−IV (trochanter to pretarsus): 257 (250−265), 221 (216−225), 215 (210−225), 242 (235−250) respectively; leg I: trochanter 24 (23−25), femur 105 (102−107), genu 58 (54−63), tibia 57 (54−59), tarsus 68 (66−70); leg II: trochanter 30 (29−33), femur 95 (92−98), genu 53 (50−55), tibia 48 (46−50), tarsus 63 (61−65); leg III: trochanter 32 (30−34), femur 74 (71−75), genu 40 (39−41), tibia 68 (66−70), tarsus 68 (66−70); leg IV: trochanter 32 (30−35), femur 95 (91−98), genu 42 (40−45), tibia 74 (71−76), tarsus 70 (69−73); chaetotaxy of legs I−IV (eupathidia and solenidia in parenthesis): coxae 2−1−1−1, trochanters 1−1−1−1, femora 5−4−2−1, genua 4−4−1−2, tibiae 6(1)−5−4−4, tarsi 12(3ζ, 2ω)−11(3ζ, 1ω)−10(1ω)−10(1ω).

**Male** (n = 2) (Figures 23–30).

Body oval; Length of body (excluding gnathosoma) 236−246, (including gnathosoma) 335−353, maximum width 154−165.

**Figure 23.**
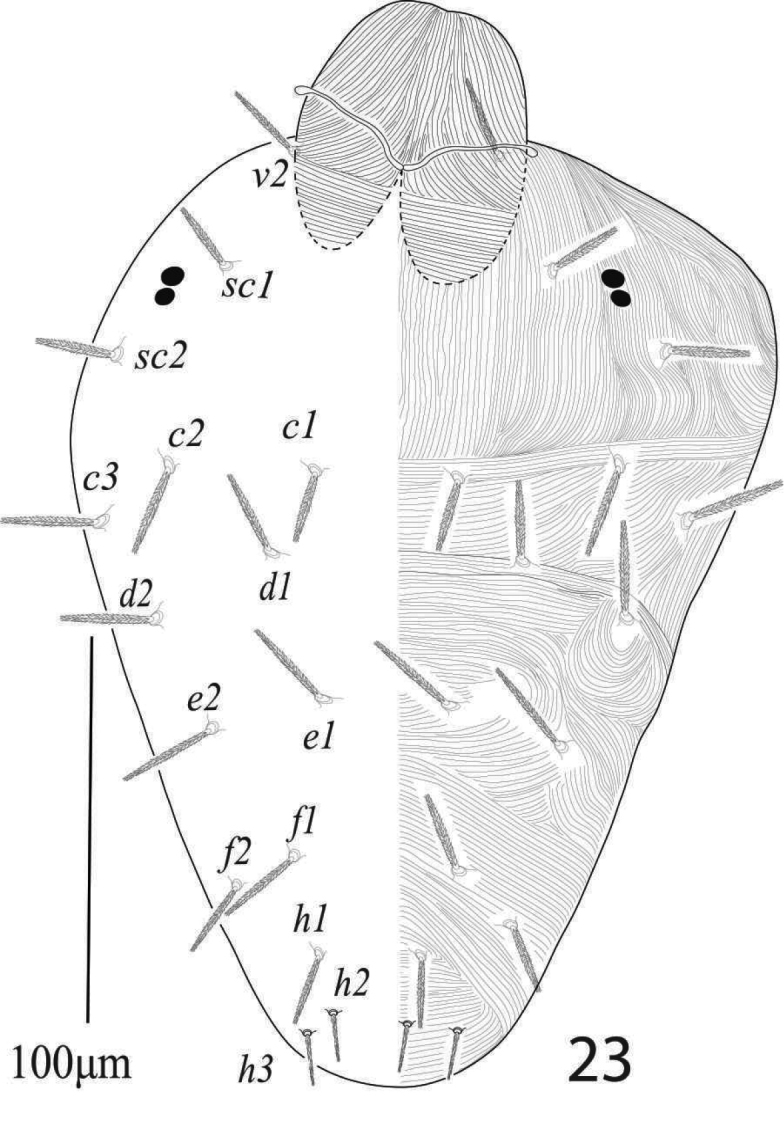
*Eutetranychusneotransversus* sp. n. Male, Dorsum.

*Dorsum* (Figure 23). Propodosoma medially with longitudinal striae; hysterosoma medially with transverse striae; all dorsal body setae slender, serrate and sub-equal in length, setae *sc2* and hysterosomal setae with small tubercles. Length of dorsal setae: *v2* 19−21, *sc1* 18−20, *sc2* 20−24, *c1* 19−22, *c2* 24−28, *c3* 21−24, *d1* 20−23, *d2* 25−27, *e1* 24−26, *e2* 20−24, *f1* 28−33, *f2* 20−23, *h1* 19−21, *h2* 9−11, *h3* 12−13; distance between dorsal setae: *v2−v2* 48−54, *sc1−sc1* 80−85, *sc2−sc2* 143−148, *c1−c1* 35−37, *c2−c2* 115−120, *c3−c3* 160−164, *d1−d1* 63−65, *d2−d2* 120−125, *e1−e1* 32−36, *e2−e2* 86−90, *f1−f1* 42−43, *f2−f2* 68−70, *h1−h1* 25−27, *h2−h2* 17−19, *h3−h3* 38−40, *v2−sc1* 32−33, *sc1−sc2* 36−38, *sc2−c3* 43−45, *sc2−c2* 32−34, *sc2−c1* 60−63, *c1−c2* 37−38, *c2−c3* 26−28, *c1−d1* 24−26, *c2−d2* 37−38, *d1−e1* 42−45, *d2−e2* 32−34, *e1−f1* 43−45, *e2−f2* 38−40, *f1−h1* 22−22, *f2−h1* 28−30.

**Figure 24.**
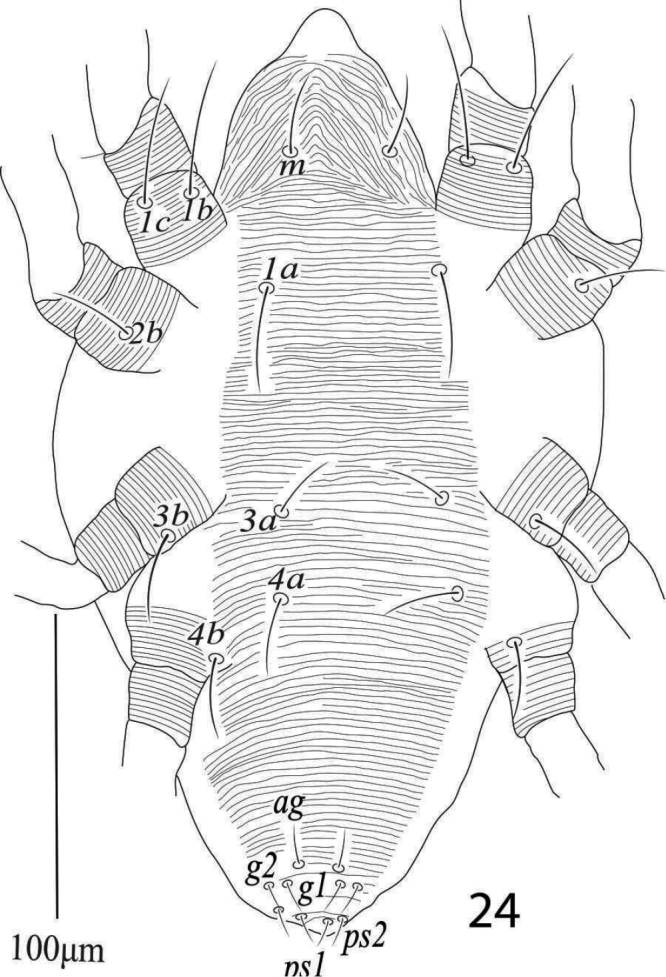
*Eutetranychusneotransversus* sp. n. Male, Venter.

*Venter* (Figure 24). Idiosoma ventrally with transverse striae from setae *1a−ag.* Length of ventral setae; *1a* 35−38, *3a* 22−24, *4a* 26−28, *1b* 45−48, *1c* 42−47, *2b* 26−30, *3b* 30−33, *4b* 27−28; distance between setae: *1a−1a* 55−58, *1b−1c* 13−16, *3a−3a* 44−47, *4a−4a* 62−65; aggenital setae: *ag* 14−16, *ag−ag* 10−11; genital setae: *g1* 10−11, *g2* 11−12, *g1−g1* 16−17, *g2−g2* 24−26; anal setae two pairs: *ps1* 8−9, *ps2* 12−13, *ps1−ps1* 8−9, *ps1−ps2* 6−7.

**Figure 25.**
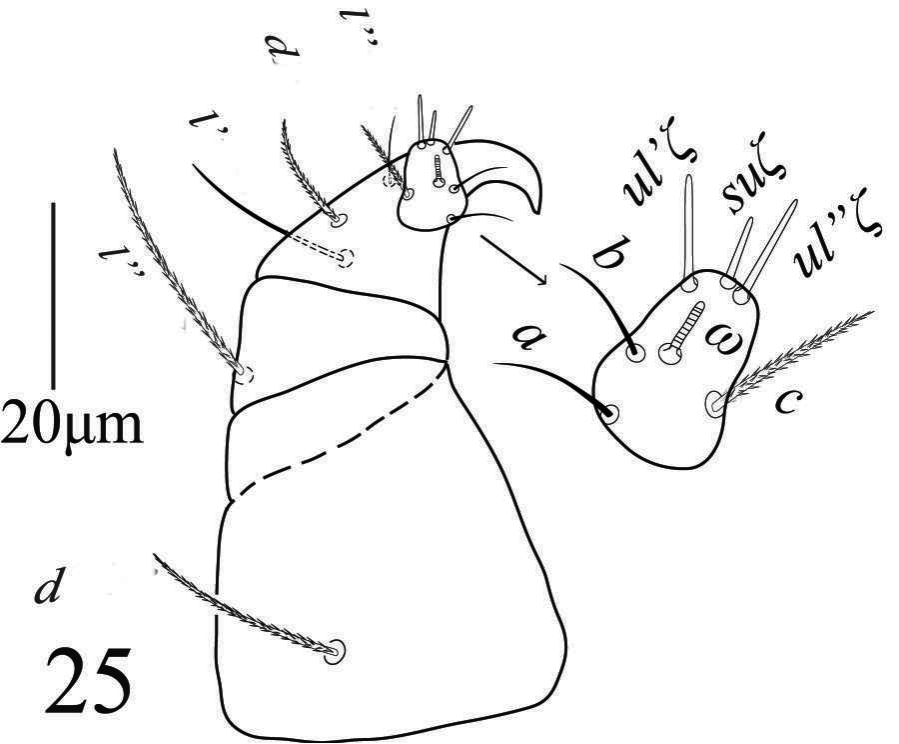
*Eutetranychusneotransversus* sp. n. Male, Palp.

*Gnathosoma* (Figure 25). Subcapitular setae *m* 24−28, *m*−*m* 30−31 (Figure 24); palp femur and genu each with one setae *d* 20−22, *l*” 26; palp tibia with three setae *d* 12−13, *l*” 17−19, *l*’ 8 and a palp tibial claw; palp tarsus 11 long, 8 wide, with 3 simple setae *a* 7−8, *b* 6, *c* 10−11, 3 eupathidia *ul*’’ζ = *ul*’ζ 6−7, width 0.7 (0.6−0.9) *su*ζ 4, 0.6 (0.5−0.7) a solenidion ω 3.5 long, width 1 (0.9−1.2) (Figure 25). Stylophore slightly notched; peritremes with simple bulb terminaly (Figure 23).

**Figure 26.**
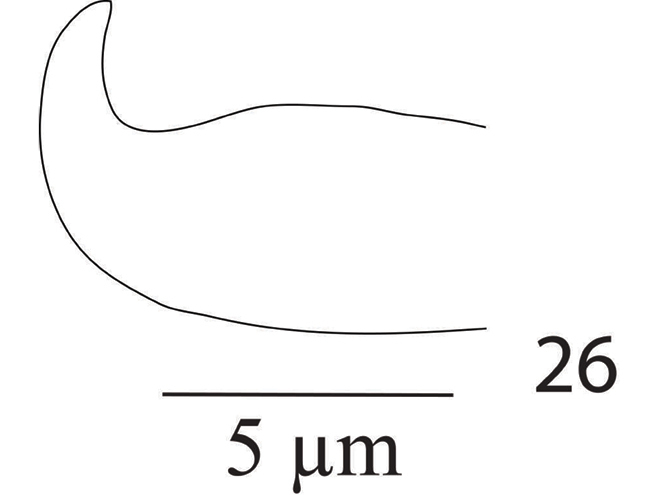
*Eutetranychusneotransversus* sp. n. Male, Aedeagus.

*Aedeagus* (Figure 26) bends dorsad at an angle of 90°; the bent portion blunt distally, shaft 8 long, 4 wide, bent portion 2.5 long.

**Figures 27–30.**
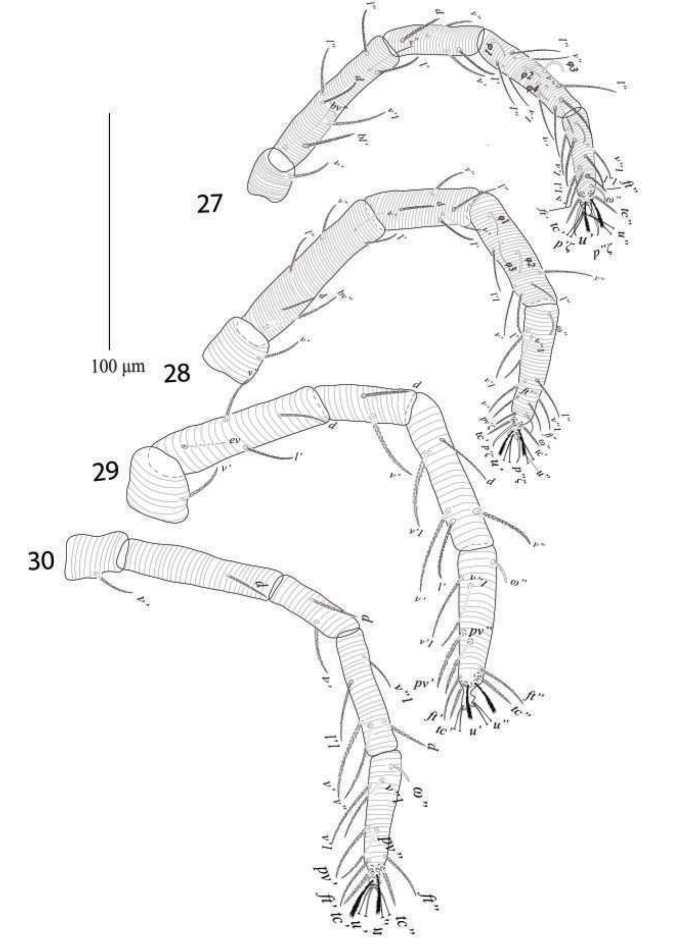
*Eutetranychusneotransversus* sp. n. Male, **27** Leg 1 **28** Leg 2 **29** Leg 3 **30** Leg 4.

*Legs* (Figures 27–30). Length of legs I−IV (trochanter to pretarsus): 315−325, 269−275, 265−271, 268−275 respectively; chaetotaxy of legs I−IV (eupathidia and solenidia in parenthesis): coxae 2−1−1−1, trochanters 1−1−1−1, femora 8−6−4−1, genua 5−5−2−2, tibiae 9(4)−5(3)−5−4, tarsi 11(2ζ, 2ω)−11(3ζ, 2ω)−10(1ω)−10(1ω).

######## Immature stages.

Unknown.

######## Etymology.

The species name is derived from the transverse striations on dorsal hysterosoma.

######## Type material.

Holotype female and four paratype females, *Juniperusprocera* Hochst. Ex Endl. (Cupressaceae), Al-Shifa road, Taif, 21°04.690'N, 040°18.928'E, elevation 2244 m, 11 Oct 2016, leg. M Kamran and M Rehman; three paratype females, *J.procera* , Ash Shifa road, Taif, 21°06.481'N, 040°20.526'E, elevation 2133 m, 12 Sept 2017, leg. Eid M Khan and M Rehman.

######## Remarks.

*Eutetranychusneotransversus* sp. n. belongs to *orientalis* species group. It closely resembles *E.transverstriatus* Smiley & Baker, 1995 because the entire hysterosoma dorsomedially in both bear transverse striations. The new species is different from *E.transverstriatus* by stylophore anteriorly slightly notched vs. rounded; hysterosomal striae without lobes vs. with distinct lobed striae; number of setae on femora I−IV 5−4−2−1 vs. 7−7−4−3; genu III 1 vs. 2 and tibiae I−IV 6(1)−5−4−4 vs. 10−6−6−6 in *E.transverstriatus* (Table 1).

####### Eutetranychus palmatus

Taxon classificationAnimaliaProstigmataTetranychidae

Attiah, 1967

Figures 31 , 32 , 33 , 34 , 35−38 , 39 , 40 , 41 , 42 , 43–46

Eutetranychus palmatus Attiah, 1967: 12−13, Meyer 1974: 137, Meyer 1987: 78, Palevsky et al. 2010: 43−51, Ben-David et al. 2013: 129.

######## Material examined.

Eight females, *Washingtonia* sp. (Arecaceae), Taif, 21°17.220'N, 040°21.963'E, elevation 1736 m, 11 Oct 2016, leg. M Kamran and M Rehman; seven females, *Washingtonia* sp., Tabuk, 28°23.754'N, 036°32.81'E.

######## Known Hosts.

Date palm, *Phoenixdactylifera* L. (Attiah 1967, Palevsky et al. 2010); the desert fan palm, *Washingtoniafilifera* Lindley, Wendland; doum palm, *Hyphaenethebaica* L. Martius; Canary Island palm, *Phoenixcanariensis* Chabaud; mountain date palm, *Phoenixloureiroi* (Ben-David et al. 2007). Alatawi (2011) misidentified specimens of *E.orientalis* as *E.palmatus* collected from *Cucurbitamoschata* Duchesne ex. Poiret (Cucurbitaceae).

######## Distribution.

Egypt, Iran, Israel, and Saudi Arabia.

######## Redescription

**of female (n = 15)** (Figures 31–38)

*Body* oval, color in life greenish yellow. Length of body (excluding gnathosoma) 414−425, (including gnathosoma) 435−455 and maximum width 325−345.

**Figure 31.**
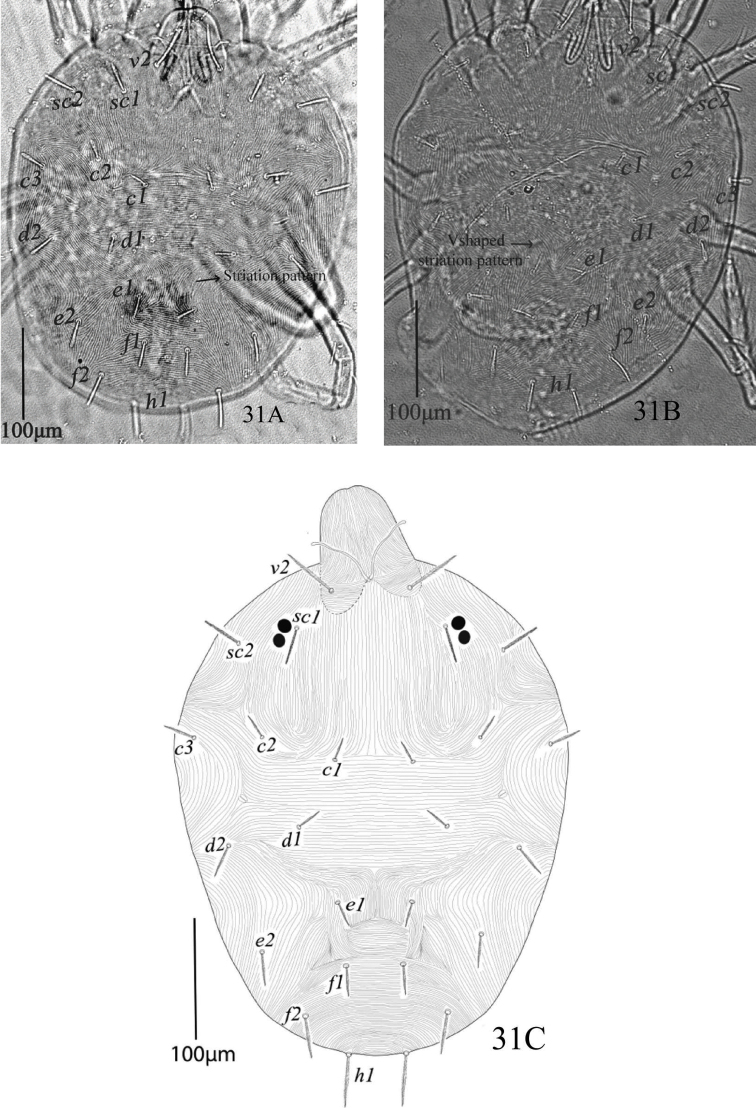
*Eutetranychuspalmatus*, Female, Dorsum (**A–C**).

*Dorsum* (Figure 31). Dorsum with lobed striae, propodosoma medially with longitudinal striae, hysterosoma medially with transverse striae except area between setae *d1* and *e1* longitudinal or “V” shaped pattern; dorsal setae serrate, slightly lanceolate, setae *c1*, *d1, e1* reaching less than half to the distance of next consecutive setae; all dorsal setae without tubercles, propodosomal setae *v2* reaching about two third to the distance *v2−v2* and reaching to the bases of setae *sc1*, setae *c3*, *d2*, *e2*, *f2*, *h1* and all propodosomal setae relatively longer than dorsocentral setae *c1*, *d1*, *e1*. Length of dorsal setae: *v2* 47−52, *sc1* 30−33, *sc2* 30−34, *c1* 17−19, *c2* 20−22, *c3* 25−29, *d1* 20−23, *d2* 28−31, *e1* 21−25, *e2* 27−32, *f1* 25−30, *f2* 32−37, *h1* 32−37; distance between dorsal setae: *v2−v2* 63−70, *sc1−sc1* 125−133, *sc2−sc2* 234−245, *c1−c1* 67−73, *c2−c2* 184−195, *c3−c3* 280−296, *d1−d1* 123−140, *d2−d2* 245−255, *e1−e1* 55−62, *e2−e2* 172−181, *f1−f1* 44−47, *f2−f2* 116−130, *h1−h1* 48−52, *v2−sc1* 40−43, *sc1−sc2* 46−50, *sc2−c3* 79−84, *sc2−c2* 72−78, *sc2−c1* 116−127, *c1−c2* 57−60, *c2−c3* 53−57, *c1−d1* 59−63, *c2−d2* 93−97, *d1−e1* 66−74, *d2−e2* 88−94, *e1−f1* 47−50, *e2−f2* 62−66, *f1−h1* 62−67, *f2−h1* 42−45.

**Figure 32.**
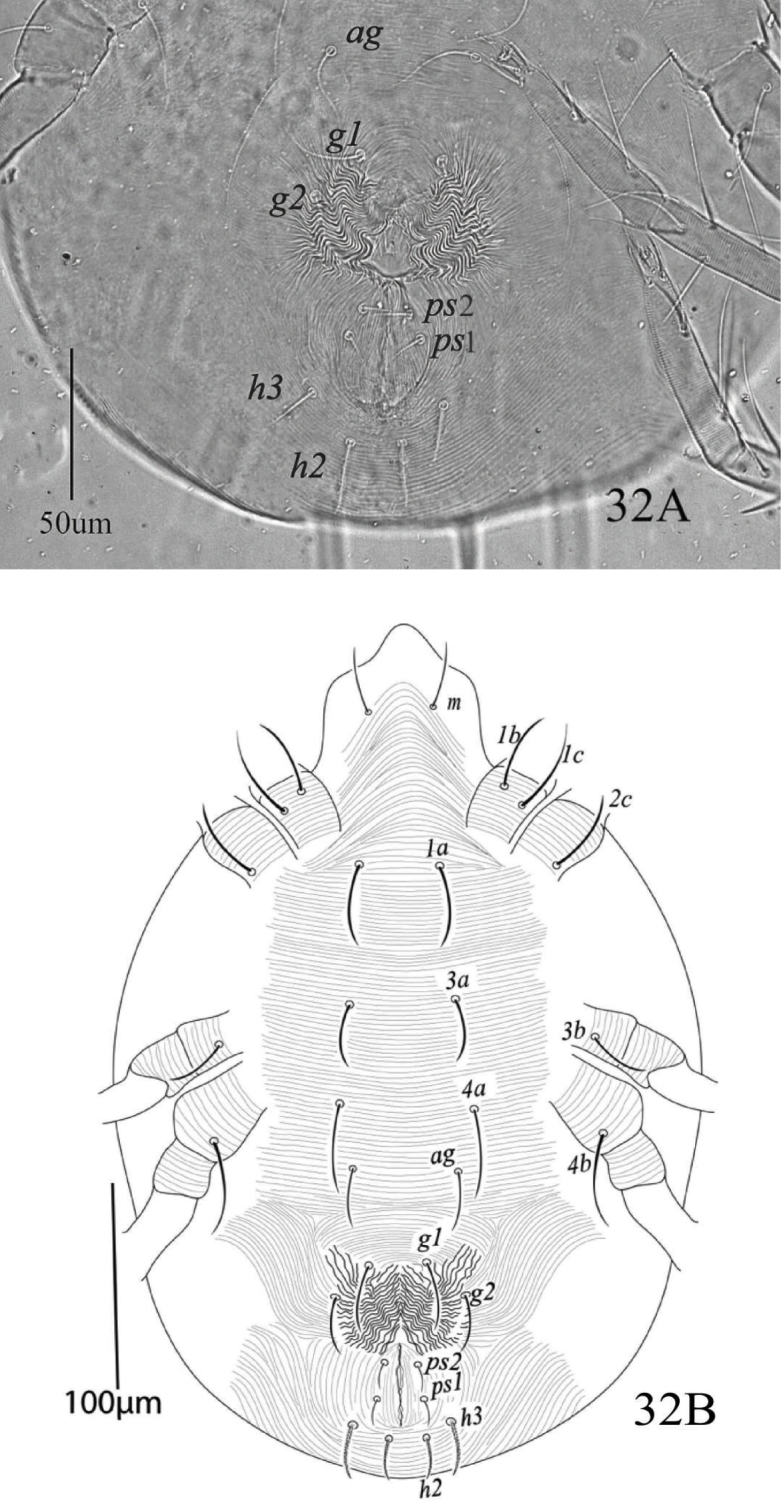
*Eutetranychuspalmatus*, Female, **A** Genito-anal region **B** Venter.

*Venter* (Figures 32, 34). Ventral integument with transverse striae between setae *1a* to *g1*. Length of ventral setae; *1a* 39−43, *3a* 30−34, *4a* 41−44, *1b* 35−39, *1c* 42−47, *2c* 36−40, *3b* 29−33, *4b* 42−46; distance between intercoxal and coxae setae: *1a−1a* 40−43, *1b−1c* 10−11), *3a−3a* 63−66, *4a−4a* 82−88; aggenital setae: *ag* 29−32, *ag−ag* 51−55; genital setae: *g1* 30−34, *g2* 26−31, *g1−g1* 28−32, *g2−g2* 61−66; anal setae two pairs: *ps1* 11−13, *ps2* 10−11, *ps1−ps1* 16−18, *ps1−ps2* 22−26; para anal setae two pairs: *h2* 18−20, *h3* 23−27, *h2−h2* 16−17, *h3−h3* 46−50, para-anal setae *h2* and *h3* finely serrated. Spermatheca oval, elongated and sacculus terminally rounded or slightly pointed as shown in figure 34.

**Figure 33.**
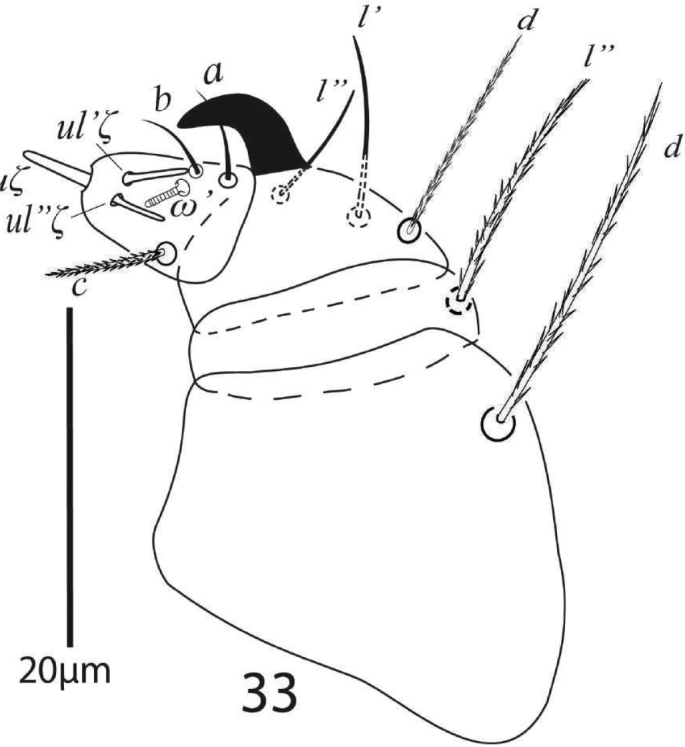
*Eutetranychuspalmatus*, Female, Palp.

*Gnathosoma* (Figure 31). Subcapitular setae *m* 30−34, *m*−*m* 37−42 (Figure 32). Palp femur and genu each with one setae *d* 45−49, *l*” 32−37; palp tibia with three setae *d* 24−27, *l*” 21−24, *l*’ 15−16 and a palp tibial claw; palp tarsus 19 long, 14 wide, with 3 simple setae *a* 10−11, *b* 9−10, *c* 13−14, 3 eupathidia *su*ζ 9, width 1.35−1.7, *ul*’’ζ = *ul*’ζ 7−8, width 1.3−1.6 a solenidion ω 5 long, width 1.9−2.2 (Figure 33). Stylophore anteriorly slightly notched; peritremes ending with simple bulb (Figure 31).

**Figure 34.**
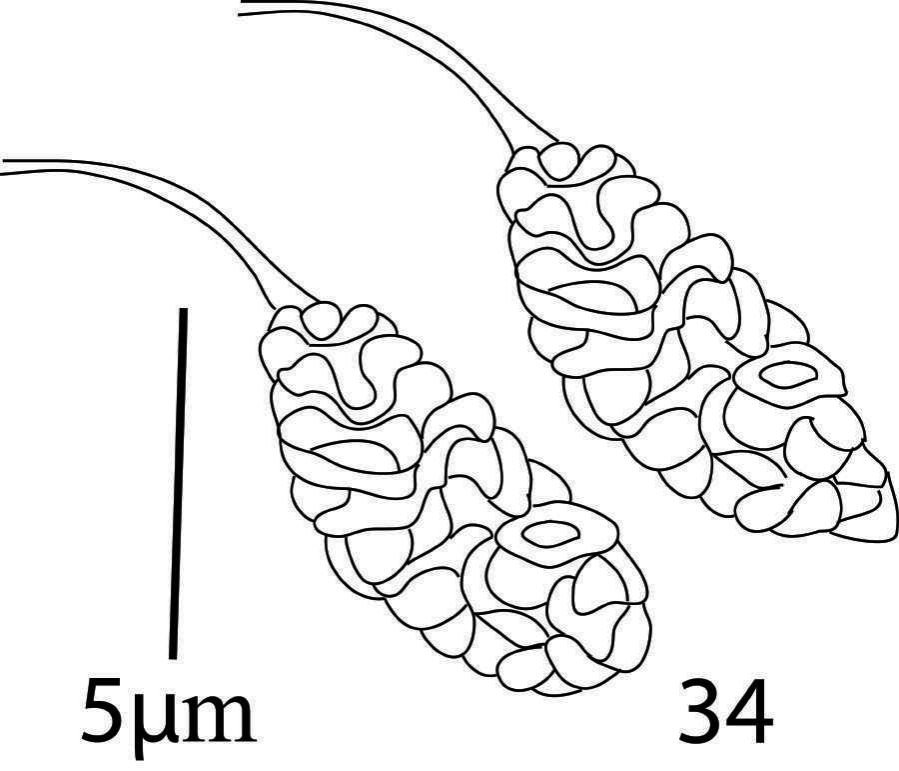
*Eutetranychuspalmatus*, Female, Spermatheca.

**Figures 35–38.**
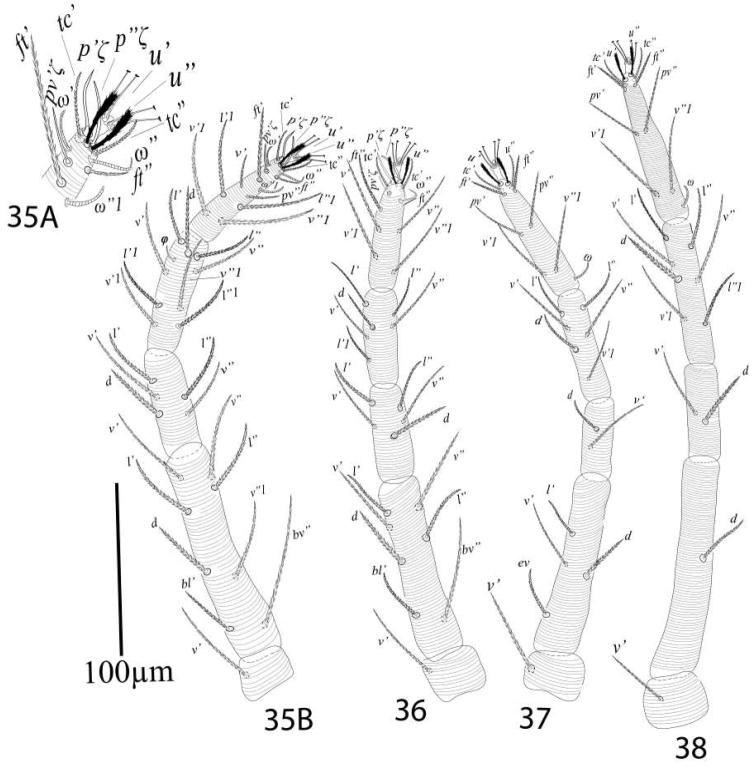
*Eutetranychuspalmatus*, Female, **35A** leg1 tarsus **35B** Leg 1 **36** Leg 2 **37** Leg 3 **38** Leg 4.

*Legs* (Figures 35–38). Length of legs I−IV (trochanter to pretarsus): 320−340, 285−300, 280−295, 335−350 respectively; leg I 320−340: trochanter 30−33, femur 112−125, genu 61−68, tibia 63−72, tarsus 61−67; leg II 285−300: trochanter 30−33, femur 88−93, genu 50−55, tibia 46−50, tarsus 67−72; leg III 280−295: trochanter 25−30, femur 95 93−99, genu 30−33, tibia 58−63, tarsus 76−80; leg IV 335—350: trochanter 25−29, femur 110−117, genu 48−55, tibia 73−79, tarsi 77−82; legs chaetotaxy I−IV (solenidia in parenthesis): coxae 2−1−1−1, trochanters 1−1−1−1; femora 8−7−4−1; genua 5−5−2−2; tibiae 9(1) −6−6−7; tarsi 12 (3ζ, 3ω) −10(3ζ, 1ω) −10(1ω) −10(1ω).

**Male** (n= 4) (Figures 39–46)

*Body* oval, length of body (excluding gnathosoma) 340−355, (including gnathosoma) 405−425 and maximum width 206−220.

**Figure 39.**
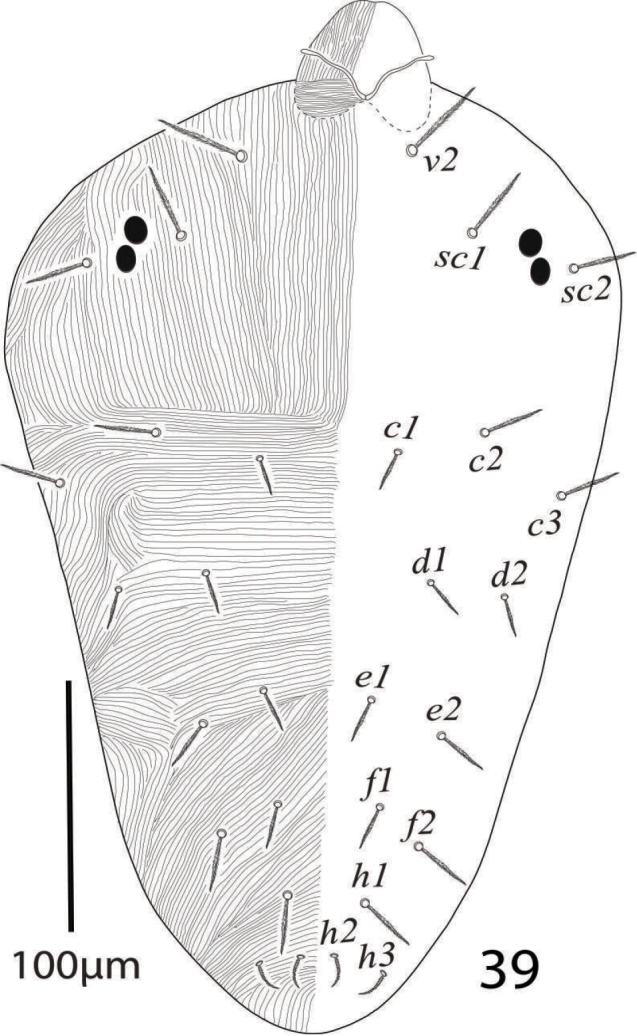
*Eutetranychuspalmatus*, Male, Dorsum.

*Dorsum* (Figure 39). Propodosoma medially with longitudinal striae, area between setae *c1*−*d1* with transverse striae and *e1*−*h1* with oblique striae; all dorsal setae short and slightly lanceolate, and without tubercles; length of dorsal setae: *v2* 30−32, *sc1* 28−33, *sc2* 21−24, *c1* 15−17, *c2* 19−22, *c3* 20−24, *d1* 14−16, *d2* 14−17, *e1* 16−19, *e2* 19−22, *f1* 16−18, *f2* 19−22, *h1* 25−28, *h2* 9−12, *h3* 11−13; distance between dorsal setae: *v2−v2* 60−68, *sc1−sc1* 90−103, *sc2−sc2* 180−195, *c1−c1* 45−52, *c2−c2* 115−125, *c3−c3* 180−196, *d1−d1* 80−89, *d2−d2* 130−142, *e1−e1* 38−44, *e2−e2* 80−89, *f1−f1* 35−40, *f2−f2* 65−70, *h1−h1* 25−30, *h2−h2* 11−13, *h3−h3* 41−48, *v2−sc1* 36−42, *sc1−sc2* 30−34, *sc2−c3* 75−84, *sc2−c2* 64−70, *sc2−c1* 86−96, *c1−c2* 38−42, *c2−c3* 33−37, *c1−d1* 45−50, *c2−d2* 55−61, *d1−e1* 46−54, *d2−e2* 55−60, *e1−f1* 39−45, *e2−f2* 38−43, *f1−h1* 32−35, *f2−h1* 25−31.

**Figure 40.**
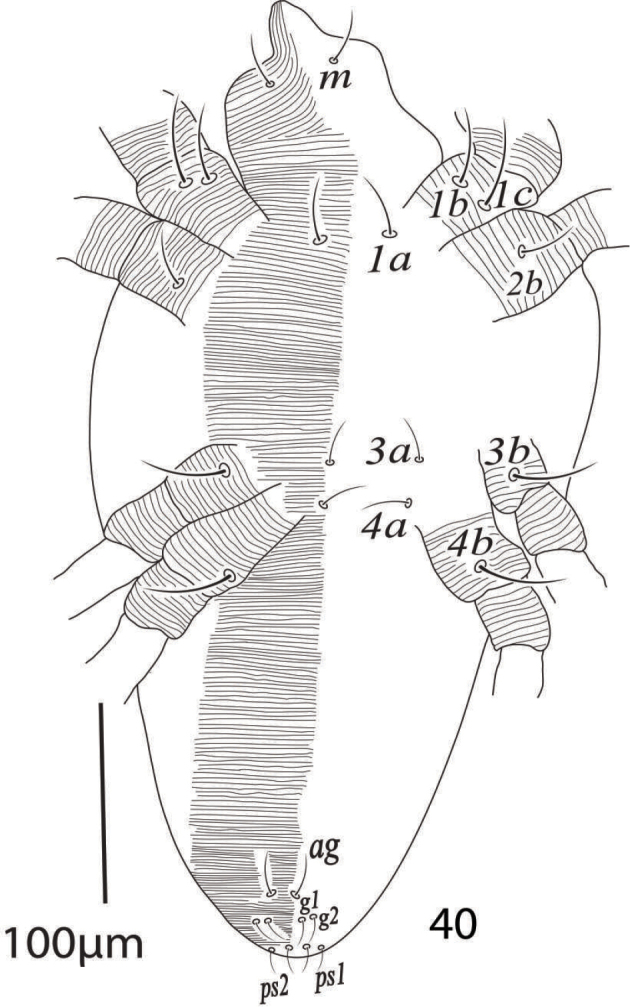
*Eutetranychuspalmatus*, Male, Venter.

*Venter* (Figure 40). Idiosoma ventrally with transverse striae; length of ventral setae; *1a* 30−32, *3a* 20−24, *4a* 24−28, *1b* 35−39, *1c* 42−47, *2b* 28−30, *3b* 39−43, *4b* 42−46, distance between setae: *1a−1a* 34−38, *1b−1c* 10−11, *3a−3a* 38−45, *4a−4a* 38−42; aggenital setae: *ag* 20−22, *ag−ag* 6−7; genital setae: *g1* 9−11, *g2* 10−11, *g1−g1* 16−17, *g2−g2* 25−28; anal setae two pairs: *ps1* 9−11, *ps2* 11−12, *ps1−ps1* 19−21, *ps1−ps2* 7.

**Figure 41.**
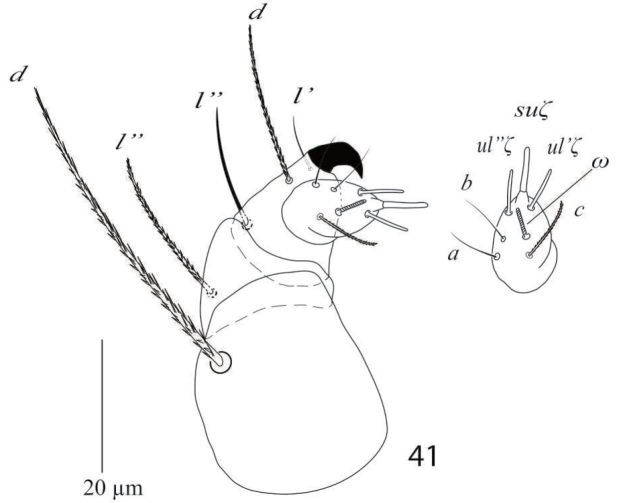
*Eutetranychuspalmatus*, Male, Palp.

*Gnathosoma* (Figure 41). Subcapitular setae *m* 27−29, *m*−*m* 30−33 (Figure 40); palp femur and genu each with one setae *de* 38−41, *l*” 21−25; palp tibia with three setae *d* 16−20, *l*” 21−25, *l*’ 13−14 and a palp tibial claw; palp tarsus 9−10 long, 12 wide, with 3 simple setae *a* 7 −8, *b* 8, *c* 9−10, 3 eupathidia *ul*’’ζ = *ul*’ζ 6.5−7, width 1 (0.9−1) *su*ζ 4, width 0.7−0.9 a solenidion ω 3 long, width 1.2−1.7 (Figure 41). Stylophore notched; peritremes ending with simple bulb (Figure 39).

**Figure 42.**
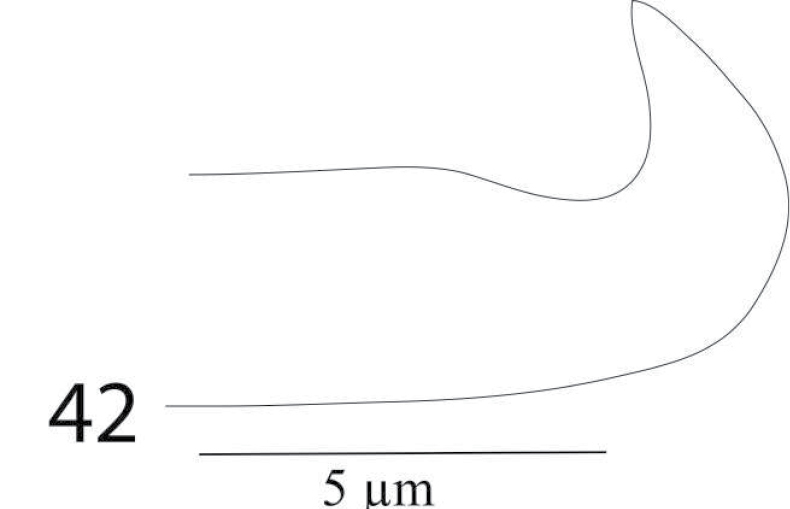
*Eutetranychuspalmatus*, Male, Aedeagus.

*Aedeagus* (Figure 42) bends dorsad at an angle of 70°; aedeagal knob pointed distally, shaft 10 long, 3.4 wide, bent portion 2.8 long.

**Figures 43–46.**
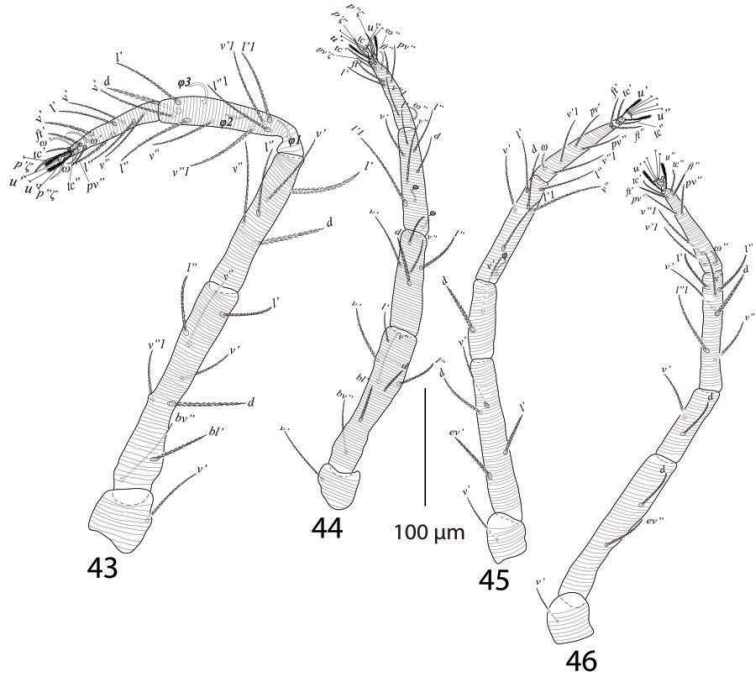
*Eutetranychuspalmatus*, Male, **43** Leg 1 **44** Leg 2 **45** Leg 3 **46** Leg 4.

*Legs* (Figures 43–46). Length of legs I−IV (trochanter to pretarsus): 470−485, 385−400, 402−425, 399−420 respectively; legs I−IV chaetotaxy (solenidia in parenthesis): coxae 2−1−1−1, trochanters 1−1−1−1; femora 8−7−4−1/2; genua 5−5−2−2; tibiae 9(3) −6(2)−6(1)−7; tarsi 12 (2ζ, 3ω)−10(3ζ, 2ω) −10(1ω)−10(1ω).

######## Remarks.

*Eutetranychuspalmatus* Attiah, 1967 is different from all other species of the genus *Eutetranychus* by having all dorsal body setae without tubercles. It was described and illustrated from date palm trees in Egypt (Attiah 1967), but its original description was briefly and incomplete i.e. leg chaetotaxy, length and distance of dorsal setae were not provided. Meyer (1987) identified some specimens as *E.palmatus* from date palms from Israel and provided legs chaetotaxy without illustrations. Chaetotaxy of *E.palmatus* specimens collected from *Washingtonia* sp. from Saudi Arabia is same as mentioned by Meyer (1987) except differences on femora II and III 7−4 vs. 6−2 in the specimens from Israel. However, femur III with three setae was illustrated in original illustrations (Attiah 1967). Moreover, Attiah (1967) and Meyer (1987) observed striations of prodorsum longitudinal and undulating in this species. Undulation in prodorsal striations usually happened during mounting is not important diagnostic character to differentiate the species of the genus *Eutetranychus*. Also, in this species striae between setae *d1* and *e1* were either longitudinal (Figure 31A) or “V” shaped (Figure 31B).

####### Eutetranychus orientalis

Taxon classificationAnimaliaProstigmataTetranychidae

(Klein)

**Figure 47.**
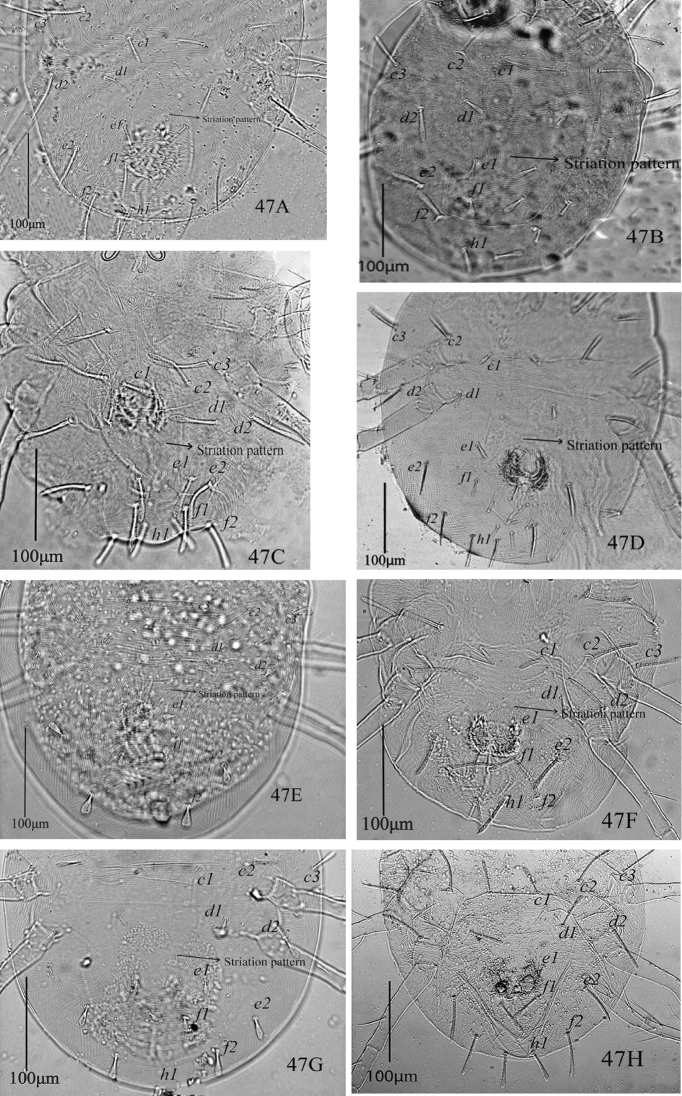
*Eutetranychusorientalis* (Klein), Females, Variation in shape of setae and striations pattern between setae *e1* on dorsum; host plants and regions, **A** Date palms, Riyadh **B** Fig, Al-Ula **C** Guava, Taif **D**Citrus, Riyadh **E** Lemon, Al-Ula **F**Citrus, Riyadh **G**Citrus, Najran **H**Citrus, Riyadh.

**Figure 48.**
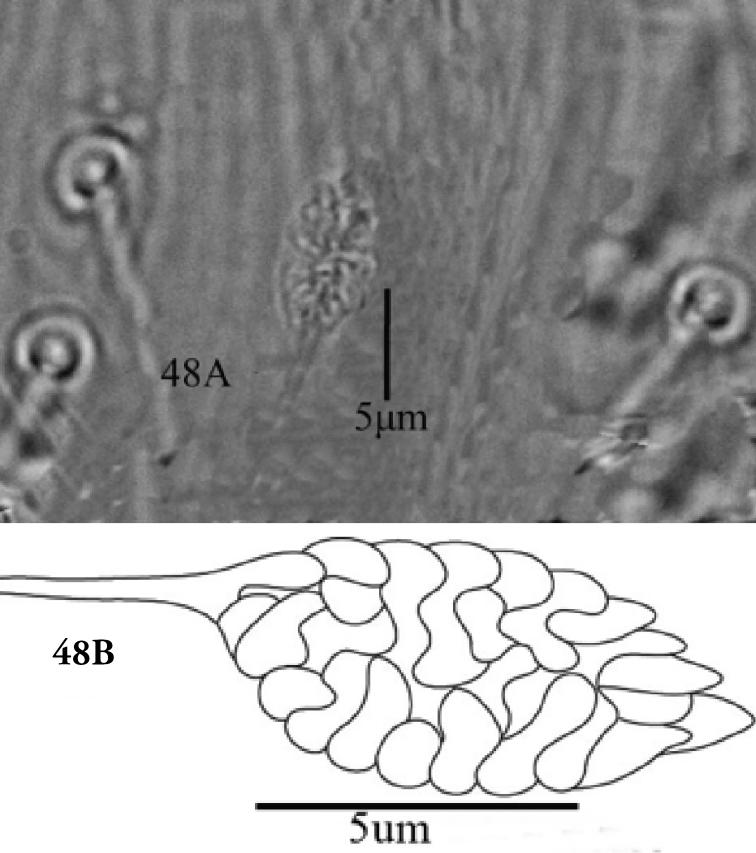
**A, B***Eutetranychusorientalis* Female, Variation in shape of spermatheca. Pointed distally.

**Figure 49.**
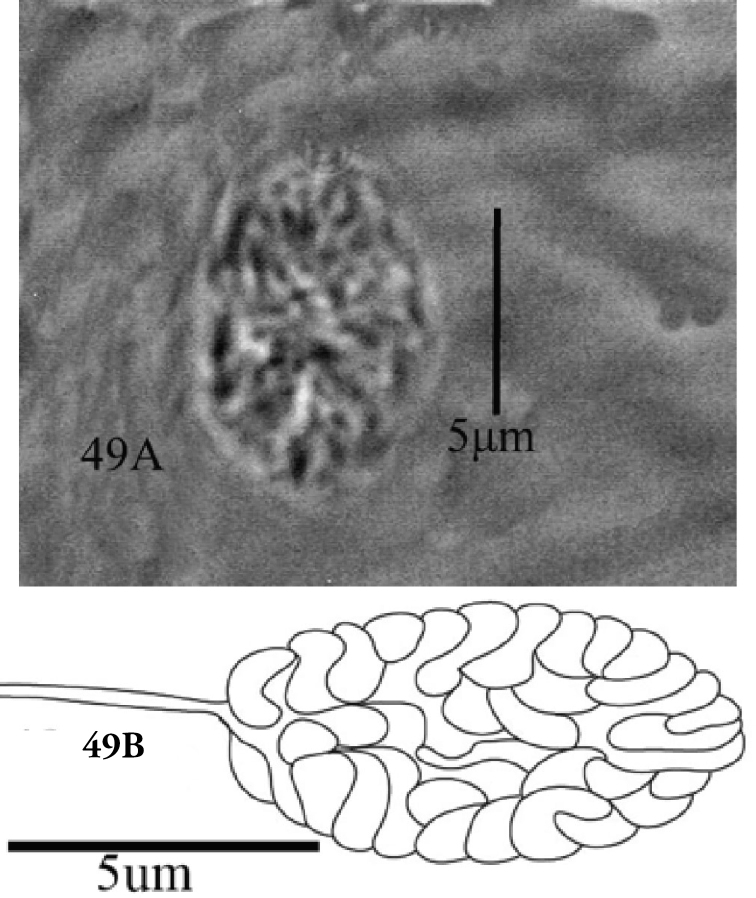
**A, B***Eutetranychusorientalis* Female, Variation in shape of spermatheca. Rounded distally.

Figures 47 , 48 , 49

Anychus latus Klein, 1936: 3.
Eutetranychus orientalis (Klein): Baker and Pritchard 1960: 464−467.
Eutetranychus monodi Andre, 1954: 859.
Eutetranychus anneckei Meyer, 1974: 148−149.
Eutetranychus sudanicus Elbadry, 1970: 301−305.

######## Previous records from Saudi Arabia.

Martin 1972, Alatawi 2011.

######## Material examined.

Twenty seven females, *Citrus* sp., Education Farm, King Saud University, Riyadh, 24°44.253'N, 46°37.225'E, 01 Feb 02 Apr 2009, 26 Oct 01 Nov 2010, 14, 24 Apr 2011, leg. J Basahih, and T Martibi; one female, *Citrus* sp., Dariyah, Riyadh, 24°44.866'N, 46°34.624'E, 02 Feb 2009, leg. J Basahih; seven females, *Vitisvinifera* and *Citrus* sp., Ammaria, Riyadh, 24°49.194'N, 46°28.163'E, 12 Apr 2009, 10 Mar 2011, leg. W Negm; five females, Hayer, Riyadh, 24°23.611'N, 46°49.464'E, 28 Apr 2009, leg. J Basahih; two females, Rhodat ul Khoraim, Riyadh, 03, 9 May 2009, leg. J Basahih;, three females, *Citrus* sp., Waseel, Riyadh, 24°48.786'N, 46°31.180'E, 11 Oct 2009, 23 Apr 2010, leg. J Basahih; four females, *Citrus* sp., *Juniperus* sp., and Grasses under *P.dactylifera*, near students housing King Saud University, Riyadh, 24°43.484'N, 46°36.985'E, 20 Sep 2010, 28 Mar 2011, leg. J Basahih; eight females, *P.dactylifera*, Imam Muhammad Ibn Saud University, Riyadh, 24°48.759'N, 46°42.735'E, 13, 27 Dec 2010, 01, 25 Jan 25, 23 Mar 2011, leg. J Basahih ; twelve females, *Citrus* sp., Nijran, 18 Apr 28 Sept 2011, leg. Jaid; six females, *Citrus* sp. Qassim, 26°00.612'N, 044°00.166'E, 26 May 2011, leg. J. Basihih and A. Majeed; two females, *Acacia* sp., and soil under *P.dactylifera* Al-Madina, 24°26.335'N, 39°36.866'E, 19 Jun 13 Oct 2011, leg. M Kamran and W Negm; eleven females, *Datura* sp., and *Citrus* sp., Wadi Namar, Riyadh, 24°34'18.9N, 46°40'40.4E, 14 Oct 2012, leg. M Kamran; two females, *Neriumoleander*, Dariyah, Riyadh, 24°44.866'N, 46°34.624'E, 5 Apr 2014, 18 Mar 2015 leg. M Kamran; two females, *Tamarix* sp. and *Saccharum* sp., Deesa valley, Tabuk, 27°36'049N, 36°25'785E, 17, 18 Oct 2015, leg. M Kamran; two females, *P.dactylifora*, Al-Sail Kabeer, Taif, 21°33.882'N, 040°18.048'E, 15 Oct 2016, leg. M Kamran and M Rehman; two females, *Citrus* sp., Khayber, 25°34.563'N, 39°19.375'E, 1 Nov 2016, leg. M Kamran and E M Khan; nine females, *Citrus* sp., *Ziziphus* sp., and *Albizia* sp., Al-Ula, 26°48.757'N, 37°58.241'E, 2 Nov 2016, leg. M Kamran and E M Khan; twenty five females, *Citrus* sp., *Mangifera* sp., *P.dactylifera*, *Olea* sp., *Psidium* sp., *Azadirachta* sp., and *Ficus* sp., Al-Ula, 26°39.923'N, 37°55.032'E. 3, 4, 5, 6, 7 May 2017, leg. E M Khan and M Rehman.

######## Discussion.

Variations within the different populations of *Eutetranychusorientalis*.

Morphological variations of *Eutetranychusorientalis* in 91 female specimens that were collected from 28 various host plants and 80 different localities in six regions of Saudi Arabia during 2009 to 2017 are shown in Figures 47A−H, 48, and 49. The lengths and shapes of dorsal body setae, striation patterns between setae *d1* and *e1*, and chaetotaxy of leg segments including femora and tibiae have been presented in Table 1.

The most prominent variations within in *E.orientalis* populations are in the length and shape of dorsal setae. These variations including, dorsocentral setae length [*c1* (10−51), *d1* (12−50), *e1* (14−41) and *f1* (10−45)] and shape [oblanceolate, ovate, obovate, subspatulate and spatulate] (Figure 47A−H). Also, these setae were either very short far behind the bases of next consecutive setae (Figure 47B, D, E, G), reaching one third to half (Figure 47A, F) or almost extending to the bases of next consecutive setae (Figure 47C, F, H). Dorsal setae *sc1, sc2, c2, c3, d2, e2, f2*, and *h1* were also varied in shape (oblanceolate, subspatulate, spatulate, slender), mostly among the specimens of different populations. The same variations in these dorsal setae have been recorded in populations of this species collected from different countries (Baker and Pritchard 1960, Chaudhri et al. 1974, Meyer 1974, Meyer 1987, Khanjani et al. 2017).

Striations patterns between the dorsocentral setae *d1* and *e1* varied either forming “V” shaped pattern (n = 80; Figure 47A-E, G, H) or a longitudinal pattern (n = 11; Figure 47F) and varied even among the specimens of the same population. Similar variations in dorsal striation patterns have also been observed by Chaudhri et al. (1974), Meyer (1987), and Khanjani et al. (2017).

Moreover, all dorsal setae in *E.orientalis* collected in this study are set on tubercles; lateral setae are on prominent tubercles as compared to dorsocentral setae (*c1*, *d1*, and *e1*) which are mostly set on relatively smaller tubercles (n = 73). However, in some specimens setae *c1*, *d1*, and *e1* are without distinct tubercles (n = 19) as shown in Figure 47D, G. This variation was observed even among the individuals of a single population collected in the current study. A similar variation has been illustrated by Chaudhri et al. (1974). However, *E.orientalis* dorsocentral setae *c1*, *d1*, and *e1* were described and illustrated only on small tubercles (Meyer 1987, Khanjani et al. 2017).

Our observations also showed that legs setal count was fixed in *E.orientalis* on coxae, trochanters, and genua I−IV (2−1−1−1, 1−1−1−1 and 5−5−2−2), respectively (see Table 1). Chaetotaxy on leg femora and tibiae were observed mostly as I−IV (8−6−3−1) and (9(1)−6−6−7), respectively. The differences in legs chaetotaxy of the specimens of *E.orientalis* belonging to the same and different populations were observed on femora I 7 (n = 3), 7/8 (n = 10); femora II 5/6 (n = 2), 6/7 (n = 2); femora III 3/4 (n = 10), 3 (n = 40); 4 (n = 23), 2/3 (n = 3); femora IV 1/2 (n = 8); on tibiae I 8/9 (1) (n = 7), 8 (1); tibia II 6/7 (n = 3); tibia III 6/5 (n = 2); tibia IV 7/6 setae (n = 2) in the current study, similar to the variations on femora and tibiae documented by Khanjani et al. (2017) in *E.orientalis* populations collected from Iran and Australia.

The spermathecal sacculus terminally varied from rounded to slightly pointed in some specimens of this study (Figures 48A, 49A). Also, the length of the spinneret on the palp tarsus varied from three to four times compared to its width. Similarly, Khanjani et al. (2017) reported that shape of spermathecal sacculus varied distally from rounded to pointed and that spinneret length also varied in *E.orientalis*. However, Meyer (1987) considered variations in shape of spermathecal sacculus (rounded or pointed distally) and length of spinneret (3 to 4 times as long as its width) as a method to differentiate *E.fici* Meyer from *E.orientalis*.

The morphological variations in *E.orientalis* have resulted in misidentifications and additions of new species in the genus *Eutetranychus*. Because some morphological variations have now been reported in *E.orientalis*, four species *Anychusricini* Rahman & Sapra, 1940, *E.monodi* André, 1954, *E.sudanicus* El Badry, 1970, and *E.annecki* Meyer, 1974 were synonymized with *E.orientalis* by Meyer (1987) and Bolland et al. (1998).

*Eutetranychusfici* Meyer, reported from Africa, was separated from *E.orientalis* by the slightly longer dorsocentral setae, shape of spermathecal sacculus, and length of palp spinneret (Table 2; Meyer 1987). The three species *E.pruni*, *E.ricinus*, and *E.sanaae* reported from Yemen were differentiated from *E.orientalis* by variation in the number of setae on femora I and IV, shapes of dorsal setae, and striation pattern between setae *d1* and *e1* (Smiley and Baker 1995). *Eutetranychusphaseoli* Nassar & Ghai, 1981 reported from India was separated from *E.orientalis* based on the difference in numbers of setae on femur I and distances between dorsal setae *e1* and *f1* (see also Table 2). The two species *E.guangdongensis* and *E.xianensis*, reported from China, were distinguished from *E.orientalis* and *E.banksi*, respectively, based only on differences in lengths of dorsal setae (Ma and Yuan 1982) (Table 2). However, the leg chaetotaxy of these two species were mentioned in the original descriptions as being similar in *E.orientalis* (Ma and Yuan 1982) (Table 2).

Because these seven species have been differentiated in their original descriptions by only one or more variable characters which have also been observed in *E.orientalis* populations (Chaudhri et al. 1974, Meyer 1987, Khanjani et al. 2017) as well as this study (see Table. 2), these seven *Eutetranychus* species (*E.phaseoli*, *E.guangdongensis*, *E.xianensis*, *E.fici*, *E.pruni*, *E.ricinus*, and *E.sanaae*) are suggested as synonyms of *E.orientalis* in this study.

**Table 2.** Variable morphological characters used to differentiate some *Eutetranychus* species suggested as synonyms of *E.orientalis* in the current study.

Suggested synonyms of *E.orientalis*	Characters used to differentiate in original descriptions							Reference
	Dorsocentral setae, short, medium or long	Shape of dorsal setae	Setae on femur II	Setae on femur IV	Striations pattern b/w *e1* and *d1*	Spermatheca distally	Length of palp spinneret as compared to width	
*** E. fici ***	long, extending to the bases of next setae in line	Spatulate to subspatulate	6	2	V shaped	rounded	4 times	Meyer 1987
*** E. pruni ***	Short to medium	Slender	7	1	longitudinal	–*	–*	Smiley and Baker 1995
*** E. ricinus ***	Short to medium	oblanceolate to subspatulate	7	2	longitudinal	–*	–	Smiley and Baker 1995
*** E. sanaae ***	Short to medium	Slender	7	1	V shaped	–*	–*	Smiley and Baker 1995
*** E. phaseoli ***	Short	Subspatulate	7	1	V shaped	–*	–*	Nassar and Ghai 1981
*** E. guangdongensis ***	Short to medium	Spatulate to Subspatulate	Mentioned the same as in *E.orientalis*			–*	–*	Ma and Yaun 1981
E.xianensis**	Short	Spatulate to Subspatulate				–*	–*	Ma and Yaun 1981
*** E. orientalis ***	Short, medium and long almost extending to the bases of next setae in line	Slender, Spatulate, subspatulate, oblanceolate	6, 6/7, 7 Variable	1, 2,1/2 Variable	V shaped or longitudinal	Pointed or rounded	3 to 4 times	Khanjani et al. 2017; Present study

*Information not available in the original descriptions **distinguished from *E.banksi* in the original description

###### Species *Inquirenda*

####### Eutetranychus papayensis

Taxon classificationAnimaliaProstigmataTetranychidae

Iqbal & Ali, 2008

Eutetranychus papayensis Iqbal & Ali, 2008: 125−130.

######## Host and Distribution.

Female, from *Caricapapaya* L. (Caricaceae), Abbottabad, Pakistan.

*Eutetranychuspapayensis* was described with coxae I−IV 2−2−2−2 (whereas they illustrate 2−2−1−1 setae) and three pairs of anal setae. Also, the empodium of this species were neither described nor illustrated. So, based on these characters together, *E.papayensis* can neither be placed in *Eutetranychus* nor even in other genera of the family Tetranychidae.The first author has informed us that type specimens of this species have been lost. Therefore, *E.papayensis* is considered as a species inquirenda.

After excluding those seven species which we suggest as synonyms and one species inquirenda, the genus *Eutetranychus* includes 28 species (including the new species described herein) and is divided into two species groups based on the number of setae (one or two) on coxae II: the species group *orientalis* has one seta on coxa II (12 species) and the species group *banksi* has two setae on coxa II (16 species). The number of setae on coxae II has been considered as a solid morphometric character founded to be strongly constant in all specimens of each *Eutetranychus* species (Pritchard and Baker 1955, Baker and Pritchard 1960, Chaudhri et al. 1974, Meyer 1974, 1987, Khanjani et al. 2017).

###### Key to the world species of the genus *Eutetranychus* (females)

**Table:** 

1	Coxa II with 2 setae	**species group *banksi* −2**
–	Coxa II with 1 seta	**species group *orientalis* −17**
2	Setae *f1* two times more widely spaced as setae *e1* or marginal in position	**3**
–	Setae *f1* equally spaced or slightly more widely spaced as setae *e1*	**5**
3	Hysterosoma with elipitical elevations in between dorsocentral setae *c1* and *e1*; dorsal setae set on strong tubercles	***cratis* Baker & Pritchard, 1960 (Congo)**
–	Hysterosoma without elipitical elevations in between dorsocentral setae *c1* and *e1*, dorsal setae set on small tubercles	**4**
4	Genua I and II with 5 setae, setae *f1* marginal in position	***anitae* Estebanes-Gonzalez & Baker, 1968 (Mexico)**
–	Genua I and II with 4 setae, setae *f1* in normal position	***banksi* (McGregor, 1914) (USA)**
5	Hysterosoma dorsomedially with transverse striations	***nomurai* Flechtmann, 1997 (Brazil)**
–	Hysterosoma dorsomedially with a band of “V” shaped or longitudinal striae between setae *d1* and *e1*	**6**
6	Genua III and IV with 1 seta	***acaciae* Miller, 1966 (Tasmania)**
–	Genua III and IV with more than 1 setae	**7**
7	Genu III with 5 setae	***concertativus* Meyer, 1974 (Namibia)**
–	Genu III with 2 or 3 setae	**8**
8	Genua III and IV with 3 or 4 setae	**9**
–	Genua III and IV with 2 setae	**12**
9	All dorsal body setae slender, much longer; dorsocentral setae *c1, e1* and *f1* reaching past bases of next consecutive setae	***spinosus* sp. n.**
–	All dorsal body setae short, oblanceolate to subspatulate, dorsocentral setae *c1, d1* and *f1* reaching at least half distance of next consecutive setae	**10**
10	Femur I with 7 setae, femur IV with 1 setae	**11**
–	Femur I with 8 setae, femur IV with 2 setae	***swazilandicus* Meyer, 1974 (South Africa)**
11	Tibia III with 7 setae	***rhusi* Meyer & Ueckermann, 1988 (South Africa)**
–	Tibia III with 8 setae	***namibianus* Meyer, 1987 (Namibia)**
12	Tibia II with 5 setae	**13**
–	Tibia II with 6 setae	**14**
13	Setae *v2* as long as to the distance *v2−v2*, setae *f2* reaching past bases of setae *h*; dorsal setae slender; all setae with tubercles	***bredini* Baker & Pritchard, 1960 (Rwanda)**
–	Setae *v2* and *f2* reaching one third to the distances *v2−v2* and *f2−h1*respectively; dorsal setae oblanceolate to subspatulate; only few opisthosomal setae set on tubercles	***clastus* Baker & Pritchard, 1960 (Congo)**
14	Tibia III with 6 setae, dorsocentral (*c1, d1, e1*) setae on prominent tubercles	***africanus* (Tucker, 1926) (South Africa)**
–	Tibia III with 5 setae, dorsocentral (*c1, d1, e1*) setae with small tubercles	**15**
15	Tibia IV with 7 setae, femur III with 3 setae	***enodes* Baker & Pritchard, 1960 (Congo)**
–	Tibia IV with 6 setae, femur III with 2 setae	**16**
16	Tarsus I with solenidion of loosly associated setae about two third as long as proximal tactile seta, tarsus II with this solenidion slightly longer than proximal tactile setae	***carinae* Meyer, 1974 (South Africa)**
–	Tarsus I with solenidion of loosly associated setae about less than half as long as proximal tactile seta, tarsus II with this solenidion about two third as long as than proximal tactile seta	***eliei* Gutierrez & Helle, 1971 (Madagascar)**
17	Entire hysterosoma dorsomedially with transverse striations	**18**
–	Hysterosoma dorsomedially in between setae *d1* and *e1* with longitudinal or “V” shaped band of striations	**19**
18	Femora I−IV with 5−4−2−1	***neotransversus* sp. n.**
–	Femora I−V with 7−7−4−3	***transverstriatus* Smiley & Baker, 1995 (Yemen)**
19	Idiosoma with none of dorsal setae set on tubercles	***palmatus* Attiah, 1967 (Egypt)**
–	Idiosoma with most of the dorsal body setae set on tubercles	**20**
20	Tibia II with 5 setae	**21**
–	Tibia II with 6/7 setae	**24**
21	Most of dorsal setae set on strong tubercles; striae on prodorsum medially tortuous forming crescentic pattern	***pyri* Attiah, 1982 (Egypt)**
–	Dorsal setae set on relatively small tubercles; striae on prodorsum medially longitudinal and lobed	**22**
22	Dorsal body setae slender tapering towards tips, most of dorsal setae longer than the distance between their base and the bases of the next consecutive setae	***pantopus*** (**Berlese, 1910) (Australia)**
–	Dorsal body setae sub-spatulate to oblanceolate with blunt tips; most of setaeespecially dorsocentrals (*c1, d1, e1, f1*) short far behind the next consecutive setae	**23**
23	Tibia I with 8 setae, all dorsal setae set on tubercles	***caricae* Nassar & Ghai, 1981 (India)**
–	Tibia I with 9 setae, setae *c1, d1, e1, f1, sc2* and *c3* without tubercles	***citri* Attiah, 1967 (Egypt)**
24	Tibia III with 5 setae	***maximae* Nassar & Ghai, 1981 (India)**
–	Tibia III usually with 6/7 setae or (sometime 5 setae on one side while on other side of tibia in same specimen of *E.orientalis*)	**25**
25	Femur II with 5 setae	**26**
–	Femur II usually with 6/7 except 5 setae on femur II in some specimens of *E.orientalis*)	**27**
26	Peritremes ending in bilobed bulb; setae *e2* short reaching half to the bases of setae *e1* and *f1*	***bilobatus* Nassar & Ghai, 1981 (India)**
–	Peritremes ending in a simple bulb-like structure; setae *e2* long reaching the bases of setae *e1* and *f1*	***nagai* Nassar & Ghai, 1981 (India)**
27	All dorsal setae long slender with tapering tips, dorsocentral setae *c1*, *e1*, *f1* crossing the bases of next consecutive setae; setae *e1* crossing the bases of *h1*	***mirpuriensis* Chaudhri, Akbar & Rasool, 1974** (**Pakistan)**
–	Most of dorsal setae oblanceolate to subspatulate; setae *e1* far behind the bases of *h1*	***orientalis* (Klein, 1936)**

## Supplementary Material

XML Treatment for Eutetranychus

XML Treatment for Eutetranychus spinosus

XML Treatment for Eutetranychus neotranversus

XML Treatment for Eutetranychus palmatus

XML Treatment for Eutetranychus orientalis

XML Treatment for Eutetranychus papayensis

## References

[B1] Alatawi FJ (2011) Phytophagous and predaceous mites associated with vegetable crops from Riyadh, Saudi Arabia. Saudi Journal of Biological Sciences 18(3): 239–246. 10.1016/j.sjbs.2011.02.004 23961130 PMC3730876

[B2] André M (1954) Tétranyque nouveau, parasite de *Cassiasiamea* lam. et *Grewiamollis* Juss. à Dakar. Bulletin de l’ Institut Francais d’ Afrique noire (ser. A) 16: 859–861.

[B3] Attiah HH (1967) The genus *Eutetranychus* in the U.A.R., with description of three new species. Bulletin de la Société Entomologique d’Égypte 51(11): 11–16.

[B4] Berlese A (1910) Lista di nuove specie e nuove generi di acari. Redia 6: 242–271.

[B5] Baker EW Pritchard AE (1960) The tetranychoid mites of Africa. Hilgardia 29(11): 455–574. 10.3733/hilg.v29n11p455

[B6] Banks N (1917) New mites, mostly economic (Arach. Acari.). Entomological News 28: 193–199.

[B7] Ben-David T Melamed S Gerson U Morin S (2007) ITS–2 sequences as barcodes for identifying and analyzing spider mites (Acari: Tetranychidae). Experimental and Applied Acarology 41: 169–181. 10.1007/s10493-007-9058-1 17347920

[B8] Ben-David T Ueckermann EA Gerson U (2013) An annotated list of the spider mites (Acari: Prostigmata: Tetranychidae) of Israel. Israel Journal of Entomology 43: 125–148.

[B9] Bolland HR Gutierrez J Flechtmann CHW (1998) World Catalogue of the spider mite family (Acari: Tetranychidae). Brill Academic Publishers, Leiden, 74–83.

[B10] Chaudhri WM Akbar S Rasool A (1974) Taxonomic studies of the mites belonging to the families Tenuipalpidae, Tetranychidae, Tuckerellidae, Caligonellidae, Stigmaeidae and Phytoseiidae. University of Agriculture Lyallpur, Pakistan, 250 pp.

[B11] Elbadry EA (1970) A new species of tetranychid mite from Sudan (Acarina: Tetranychidae). Revue de zoologie et de botanique africaines 82(3–4): 301–305.

[B12] Estebanes-Gonzalez ML Baker EW (1968) Ara-as rojas de Mexico (Acarina: Tetranychidae). Anales de la Escuela Nacional de Ciencias Biologicas 15: 61–133.

[B13] Flechtmann CHW (1997) Mites (Arthropoda: Acari) associated of palms (Arecaceae) in Brazil. III. *Eutetranychusnomurai* n. sp. (Tetranychidae) from Attalea phalera Ta Mart. International Journal of Acarology 23(4): 269–273. 10.1080/01647959708683576

[B14] Gerson U Venezian A Blumberg D (1983) Phytophagous mites on date palms in Israel. Fruits 38(2): 133–135.

[B15] Gutierrez J Helle W (1971) Deux nouvelles espèces du genre *Eutetranychus Banks* (Acariens: Tetranychidae) vivant sur plantes cultivées à Madagascar. Entomologische Berichten Amsterdam 31: 45–60.

[B16] Iqbal I Ali A (2008) Red spider mites from fruit orchards of Abbottabad, including a new species. Biologia 54(2): 125–130.

[B17] Jeppson LR Keifer HH Baker EW (1975) Mites Injurious to Economic Plants. University of California Press, Berkeley, 614 pp.

[B18] Kamali K (1990) A checklist of plant mites (Acari) of Khuzestan, Southwestern Iran. Scientific Journal of Agriculture 13(13): 73–83.

[B19] Khanjani M Khanjani M Seeman OD (2017) New spider mites (Acari: Tetranychidae) of the genera *Paraplonobia* and *Eurytetranychus* from Iran, and a description of all life stages of *Eutetranychusorientalis* (Klein). Acarologia 57(3): 465–491.

[B20] Klein HZ (1936) Contribution to the knowledge of the red spiders in Palestine. Bulletin Israel Agriculture Research Station 21: 1–63.

[B21] Lindquist EE (1985) Chapter 1.1.1 External anatomy, Phylogeny and Systematics. In: Helle W Sabelis MW (Eds) Spider Mites: their biology, natural enemies and control.Elsevier Science Publishers, Amsterdam, 3–28.

[B22] Meyer MKPS (1974) A revision of the Tetranychidae of Africa (Acari) with a key to the genera of the world. Entomology Memoir, Department of Agricultural Technical Services, Republic of South Africa 36: 1–291.

[B23] Meyer MKPS (1987) African Tetranychidae (Acari: Prostigmata) with reference to the world genera. Entomology Memoir, Department of Agriculture and Water Supply, Republic of South Africa, 175 pp.

[B24] Meyer MKPS Ueckermann EA (1988) South African Acari. III. On the mites of the Mountain Zebra National Park. Koedoe 31: 1–29.

[B25] Ma EP Yuan YL (1982) A new genus and five new species of Tetranychidae from China (Acari: Tetranychidae). Entomotaxonomia 4(1–2): 109–114.

[B26] Martin H (1972) Report to the Government of Saudi Arabia on Research in Plant Protection. Saudi Arabia: FAO, Rome, 1–38. [Report AGT: T/207]

[B27] Mattos VM Feres RJF (2009) Morphological pattern and life cycle of *Eutetranychusbanksi* (Acari: Tetranychidae) from different localities and hosts. Zoologia. (Curitiba) 26(3): 427–442. 10.1590/S1984-46702009000300007

[B28] McGregor EA (1914) Four new Tetranychids. Annals of the Entomological Society of America 7: 354–364. 10.1093/aesa/7.4.354

[B29] McGregor EA (1919) The red spiders of America and a few European species likely to be introduced. Proceedings of the U. S. National Museum 56: 641–679. 10.5479/si.00963801.56-2303.641

[B30] McGregor EA (1950) Mites of the family Tetranychidae. American Midland Naturalist 44(2): 257–420. 10.2307/2421963

[B31] Migeon A Dorkeld F (2006–2017) Spider Mites Web: a comprehensive database for the Tetranychidae http://www.montpellier.inra.fr/CBGP/spmweb.

[B32] Miller LW (1966) The tetranychid mites of Tasmania. Papers and Proceedings of the Royal Society of Tasmania 100: 53–76.

[B33] Nassar OA Ghai S (1981) Taxonomic studies on tetranychoid mites infesting vegetables and fruit crops in Delhi and surrounding areas. Oriental Insect 15(4): 333–396. 10.1080/00305316.1981.10434337

[B34] Palevsky E Lotan A Gerson U (2010) Evaluation of *Eutetranychuspalmatus* (Acari: Tetranychidae) as a pest of date palms in Israel. Israel Journal of Plant Science 58: 43–51. 10.1560/IJPS.58.1.43

[B35] Pritchard AE Baker EW (1955) A revision of the spider mite family Tetranychidae. The Pacific Coast Entomological Society 2: 1–472. 10.5962/bhl.title.150852

[B36] Reck GF (1959) Identification of Tetranychoid mites. Fauna Transcaucasica. Akademiya Nauk Gruzinskoi SSR Institut Zoologii Tbilisi 1: 1–150.

[B37] Rehman KA Sapra AN (1940) Mites of the family Tetranychidae from Layallpur with description of four new species. Proceeding of the Indian Academy of Science (ser. B) 11: 17–196.

[B38] Saito Y (2010) Plant mites and sociality: diversity and evolution. Tokyo, Springer, 5–38. 10.1007/978-4-431-99456-5_2

[B39] Smiley RL Baker EW (1995) A report on some tetranychid mites (Acari: Prostigmata) from Yemen. International Journal of Acarology 21(3): 135–164. 10.1080/01647959508684055

[B40] Tucker RWE (1926) Some South African mites, mainly Tetranychidae and Eriophyidae. South African Department of Agriculture Division of Entomology Memories 5: 1–15.

[B41] Vacante V (2010) Citrus Mites: Identification, Bionomy and Control. Cabi Publication, London, 197–217.

